# Energy Platform for Directed Charge Transfer in the Cascade Z‐Scheme Heterojunction: CO_2_ Photoreduction without a Cocatalyst

**DOI:** 10.1002/anie.202106929

**Published:** 2021-08-13

**Authors:** Ji Bian, Ziqing Zhang, Jiannan Feng, Madasamy Thangamuthu, Fan Yang, Ling Sun, Zhijun Li, Yang Qu, Dongyan Tang, Zewei Lin, Fuquan Bai, Junwang Tang, Liqiang Jing

**Affiliations:** ^1^ Department Key Laboratory of Functional Inorganic Materials Chemistry (Ministry of Education) School of Chemistry and Materials Science International Joint Research Center and Lab for Catalytic Technology Heilongjiang University Harbin 150080 P. R. China; ^2^ School of Chemistry and Chemical Engineering Harbin Institute of Technology Harbin 150001 P. R. China; ^3^ Department of Chemical Engineering University College London Torrington Place London WC1E 7JE UK; ^4^ International Joint Research Laboratory of Nano-Micro Architecture Chemistry Institute of Theoretical Chemistry and College of Chemistry Jilin University Changchun 130021 P. R. China

**Keywords:** charge lifetime, charge modulation, heterojunctions, photocatalysis, Z-Scheme heterojunctions

## Abstract

A universal strategy is developed to construct a cascade Z‐Scheme system, in which an effective energy platform is the core to direct charge transfer and separation, blocking the unexpected type‐II charge transfer pathway. The dimension‐matched (001)TiO_2_‐g‐C_3_N_4_/BiVO_4_ nanosheet heterojunction (T‐CN/BVNS) is the first such model. The optimized cascade Z‐Scheme exhibits ≈19‐fold photoactivity improvement for CO_2_ reduction to CO in the absence of cocatalysts and costly sacrificial agents under visible‐light irradiation, compared with BVNS, which is also superior to other reported Z‐Scheme systems even with noble metals as mediators. The experimental results and DFT calculations based on van der Waals structural models on the ultrafast timescale reveal that the introduced T as the platform prolongs the lifetimes of spatially separated electrons and holes and does not compromise their reduction and oxidation potentials.

## Graphical abstract

A cascade Z‐Scheme system composed of a dimension‐matched (001)TiO_2_‐g‐C_3_N_4_/BiVO_4_ heterojunction is proposed, in which the energy platform (001)TiO_2_ is used to direct charge transfer and separation, thus blocking the unexpected type‐II charge transfer pathway. The role of TiO_2_ is thus not only to facilitate the Z‐Scheme charge transfer but also to maintain the strong redox potentials for CO_2_ photoreduction and water splitting.

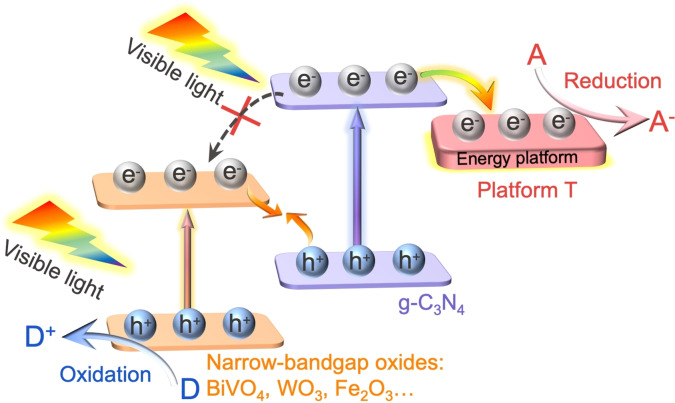

## Introduction

The severe energy crisis and environmental problems caused by the large‐scale depletion of fossil fuels is leading to a shortage of non‐renewable resources and global warming. To address these issues, the development of sustainable energy technologies is extremely essential. Photocatalysis is an appropriate technique to produce alternative green energy using highly sustainable solar energy and earth‐abundant feedstocks such as water, CO_2_, etc.[^1^, ^2^, ^3^] The rational design and fabrication of efficient and robust photocatalysts are key elements in photocatalysis and the activity is mainly controlled by the charge separation and transfer.[^4^, ^5^] So far, diverse strategies such as element doping, defect engineering, noble metal loading, and heterojunction construction, etc. have been exploited up to improve the charge carrier separation.[^6^, ^7^, ^8^] Especially, the fabrication of Z‐Scheme heterojunction nanocomposite is highly focused to enhance the charge separation and transfer as it mimics the natural photosynthesis.[^9^, ^10^, ^11^] It offers the extended choice of semiconducting materials to absorb visible light of the solar spectrum efficiently, and enhance the charge separation.^[12]^ Typically, a Z‐Scheme heterojunction is composed of photosystem I (PS I) with negative conduction band (CB) photocatalysts (i.e. g‐C_3_N_4_, CdS, and Cu_2_O) for reduction half‐reaction and photosystem II (PS II) with positive valence band (VB) photocatalysts (i.e. BiVO_4_, Fe_2_O_3,_ and WO_3_) for oxidation half‐reaction. Such artificial Z‐Scheme heterostructures (from indirect to direct) have been studied for CO_2_ reduction, water splitting, nitrogen fixation, and so on.[^13^, ^14^, ^15^] However, solar to fuel conversion efficiency is still very moderate to compete with conventional fuel production technologies. Thus, advanced strategies are highly required to design and fabricate a challenging Z‐Scheme heterojunction nanocomposite to enhance efficiency by improving charge separation and transfer.

Indeed, the photogenerated charge carrier separation from both reduction and oxidation photocatalysts and their successful transfer at the interfaces influence the Z‐Scheme performance. Recently, two‐dimension (2D) nanomaterials with sheet‐like structures have attracted significant attention to enhance charge separation and to reduce the diffusion length of charge carriers in addition to their large specific surface area.[^16^, ^17^] The construction of a robust interface between PS I and PS II using dimension‐matched 2D/2D semiconductors is facile and efficient as it offers a large contact area, less crystal boundary, and rapid charge transfer and separation channels.[^18^, ^19^] The abundant surface hydroxyl groups present on the 2D nanomaterial inspired us to construct an advanced 2D/2D Z‐Scheme heterojunction nanocomposite using the hydroxyl induced assembly process. Thus, we have fabricated the closely‐connected 2D/2D interfaces with chemical interactions to effectively facilitate the Z‐Scheme charge transfer. The artificial Z‐Scheme heterojunction features the spatial separation of photogenerated electrons and holes, while in parallel the electrons transfer from one photocatalyst with a more negative CB to the other with a less negative CB is inevitable, accompanied by the holes transfer from a more positive VB to a less positive VB, resembling what happens in the conventional type‐II heterojunction. This usually favors the charge separation with the cost of sacrificing the redox potentials of the photogenerated charge carriers. Although some groups attempted to inhibit these two competitive charge transfer pathways by designing Z‐Scheme heterojunction, still it is ambiguous.^[9]^ Hence, it is urgent and significant to find a novel strategy to maintain the redox potential as strong as possible and to prolong the lifetimes of the spatially separated charge carriers in a Z‐Scheme.

The modification of PS I semiconductor by noble metals as co‐catalysts could induce photoelectrons migrating to the surface of the co‐catalysts, which facilitates the catalytic reaction kinetics.^[20]^ Although, the charge recombination is diminished to some extent, the reduction potential of the separated electrons was dramatically mitigated due to the low Fermi level of noble metals.^[21]^ In addition, noble metals are expensive, which restricts their extensive utilization. To pump out the electrons with enough thermodynamic energy in the CB of PS I semiconductor along with a longer lifetime are the key to strengthen the Z‐Scheme charge transfer and hence improve the photoactivities. Interestingly, it has been evidenced that a wide band gap semiconductor like TiO_2_ could be taken as a proper energy platform for accepting the electrons of g‐C_3_N_4_ to prolong the charge carrier lifetime.^[22]^ Furthermore, the CB potential of the TiO_2_ nanosheet is negative enough to reduce proton, CO_2_, etc.^[10]^ Such a strategy has great prospective to enhance the Z‐Scheme charge transfer and separation by introducing an appropriate thermodynamic energy‐platform to PS I semiconductor. As this concept is relatively new, only limited experimental works have been reported so far. On the other hand, theoretical studies have to be advanced to clearly explain the Z‐Scheme charge transfer mechanism. For instance, Peng et al. studied the Z‐Scheme charge transfer mechanism using effective mass calculation, and the recombination factors were calculated to reflect the charge separation efficiency to predict the charge transfer direction.[^23^, ^24^] However, to reveal the competitive charge injection mechanism in Z‐Scheme, as discussed above similar to conventional type II, using time threshold view, in theory, remains unclear. Therefore, we endeavored here to clarify the impact of the proposed‐above energy platform on Z‐Scheme charge transfer using both experiments as well as theoretical simulation.

We have chosen a g‐C_3_N_4_ for reduction half‐reaction (PS I) as it has an inherent 2D structure, which is easy to realize the controlled fabrication of g‐C_3_N_4_ nanosheets (CN) and to minimize the diffusion distance of charge carriers inhibiting the charge recombination. On the other hand, the 2D‐BiVO_4_ nanosheet opts for oxidation half‐reaction (PS II). By combining 2D‐BiVO_4_ nanosheet (BVNS) with CN, we have realized an efficient Z‐Scheme heterojunction. With that, we have developed an original cascade Z‐Scheme system by introducing an energy platform (001)TiO_2_ on g‐C_3_N_4_ to modulate the Z‐Scheme charge transfer and separation for efficient artificial photosynthesis. This cascade Z‐Scheme system is denoted as (001)TiO_2_‐g‐C_3_N_4_/BiVO_4_ nanosheet heterojunction (T‐CN/BVNS). The optimized nanocomposite exhibits exceptional photocatalytic activities for both CO_2_ photoreduction without any cocatalysts and pure water splitting. It is verified by the experimental results and theoretical calculations that the introduced TiO_2_ could not only prolong the lifetimes of spatially separated electrons but also maintain the strong reduction potential, thus undertaking the reduction reaction on the surface of the TiO_2_ efficiently. The proposed original strategy is also suitable for other Z‐Scheme heterojunctions using other wide‐band gap oxides as an electron‐energy platform. This work not only demonstrates the introduction of an energy‐platform to promote the Z‐Scheme charge transfer but also paves avenues to synthesis cascade Z‐Scheme heterojunctions for efficient solar‐to‐fuel conversion.

## Results and Discussion

The introduction of an energy platform, e.g. a wide band gap oxide TiO_2_ or SnO_2_ layer into the Z‐Scheme would effectively prolong the photogenerated electron lifetimes of PS I semiconductors and also maintain a strong reduction potential. The classic (001)‐facet‐exposed TiO_2_ nanosheets (T) could not only meet the requirements of energy level matching for water reduction but also match the dimension of layered CN, aiming to achieve an efficient charge transfer and separation. Particularly, the low atomic coordination numbers of exposed atoms, a high density of active unsaturated coordination Ti atoms, and active surface oxygen atoms with wide bond angles of Ti‐O‐Ti in the {001} facets endow the T with higher surface energy, which is more efficient for dissociative adsorption of reactant molecules.[^25^, ^26^, ^27^]

**Figure 1:**
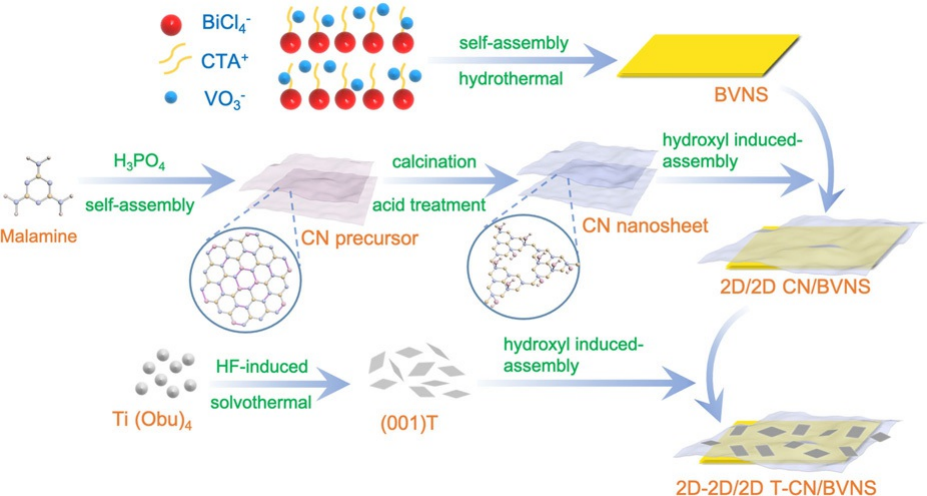
Schematic illustration of the synthesis of the designed 2D T‐2D CN/2D BVNS heterojunction.

The design and synthesis procedure of T‐CN/BVNS (2D‐2D/2D) heterojunctions are illustrated in Figure 1. The BVNS was firstly synthesized by a facile CTAB induced assembly process.^[16]^ While the CN with abundant hydroxyl groups was obtained via the acid treatment to the precursor that was synthesized through the self‐assembly of melamine and its hydrolyzed product (cyanuric acid), and the T was synthesized by an HF‐modulated solvothermal method. Through the surface hydroxyl‐induced process, the as‐prepared BVNS and CN were integrated initially, followed by the second step of T assembly on CN to form the dimension‐matched cascade Z‐Scheme T‐CN/BVNS heterojunctions.

**Figure 2:**
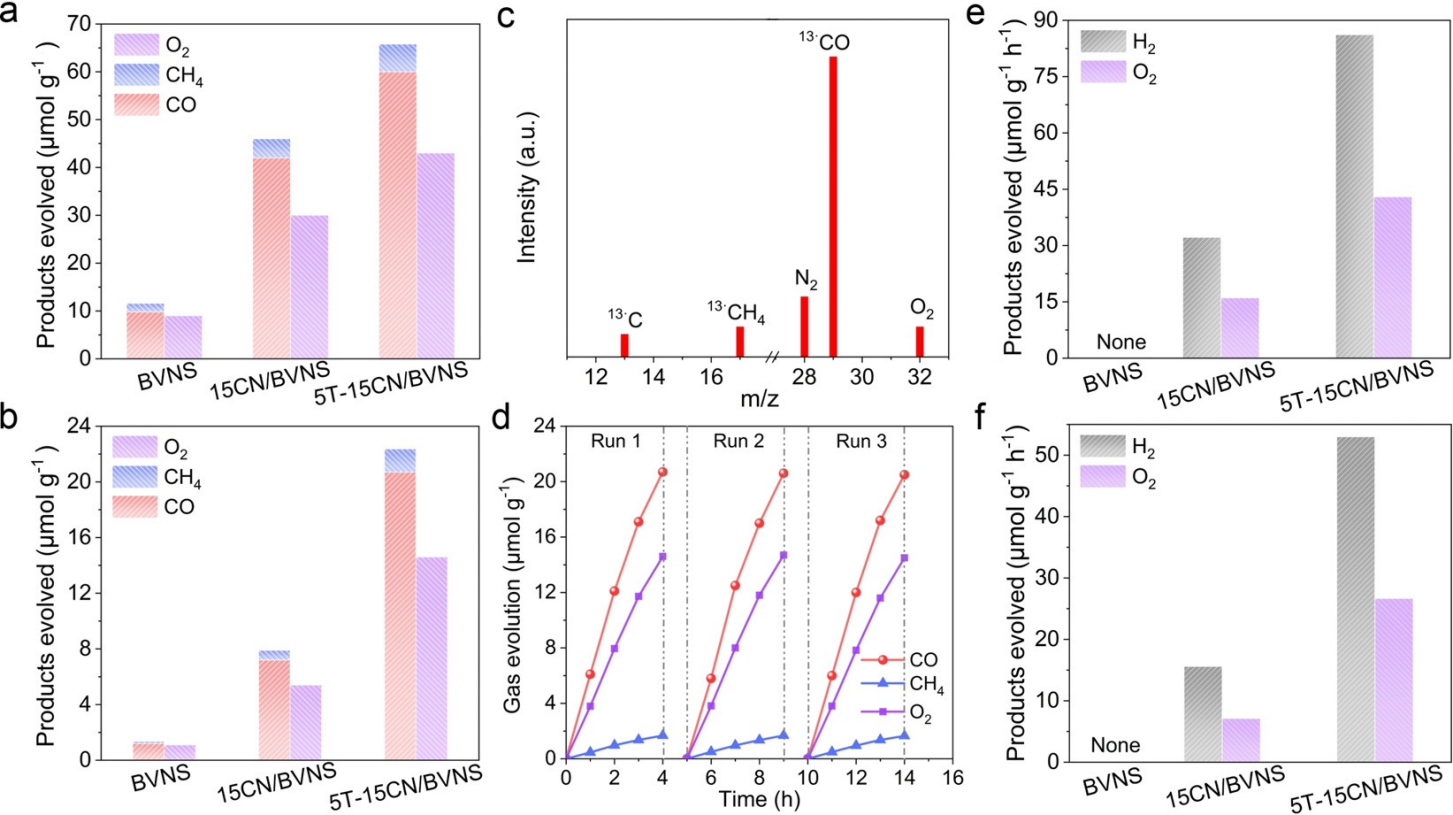
Photocatalytic activities for CO_2_ reduction under a) UV‐visible light and b) visible‐light irradiation for 4 h of BVNS, 15CN/BVNS, and 5T‐15CN/BVNS. c) Mass spectra of the products from the photocatalytic reduction of ^13^CO_2_ over 5T‐15CN/BVNS. d) Three consecutive runs of CO_2_ reduction by 5T‐15CN/BVNS under visible‐light irradiation. Photocatalytic activities for overall water splitting under e) UV‐visible light and f) visible‐light irradiation of BVNS, 15CN/BVNS, and 5T‐15CN/BVNS. The overall water splitting was carried out with Pt as the cocatalyst and without any sacrificial agents.

The photocatalytic activities of CO_2_ conversion on BVNS, *x*CN/BVNS and *y*T‐15CN/BVNS heterojunctions were investigated firstly (*x* and *y* represent the mass percentage of CN and T to BVNS, respectively). The possible products, e.g. CO CH_4_, methanol, formaldehyde, formic acid, etc. were analyzed by both gas chromatography and liquid chromatography. It shows that there are not any liquid products detected apart from the major gaseous products of CO and CH_4_. It is noticed that the photocatalytic activities for CN/BVNS heterojunctions first increases and then decreases with the loading amount of CN sheets from 10 wt % to 20 wt %, and the 15CN/BVNS is the proved best option (Figure S1). The photocatalytic activity of 15CN/BVNS is much higher than that of BVNS, CN, and 5T/BVNS (Figure S2). Similarly, the activity of the T‐CN/BVNS heterojunctions firstly increases and then decreases with the loading amount of T from 3 wt. % to 7 wt. %, in which the 5T‐15CN/BVNS delivers the best photoactivities under UV‐visible light, achieving ≈1.5‐ and ≈6‐fold enhanced photocatalytic activity compared with 15CN/BVNS and BVNS, respectively (Figure 2 a and Figure S3). A certain amount of oxidation product (O_2_) was also detected upon CO_2_ reduction. Under visible light irradiation, the photocatalytic activities of the investigated samples are not so high as those observed under UV‐visible light irradiation. However, the photocatalytic activity still increase by ≈3‐fold and ≈19‐fold compared with those of 15CN/BVNS and pristine BVNS (Figure 2 b). Further, the ^13^C isotopic experiment confirms the carbon source in the products. ^13^CO (*m*/*z*=29) and ^13^CH_4_ (*m*/*z*=17) as the major products (Figure 2 c and Figure S4) clearly indicates that they indeed originate from the conversion of CO_2_, rather than the decomposition of CN. The optimized 5T‐15CN/BVNS delivers a similar CO_2_ conversion rate across three runs, revealing the high stability of the photocatalyst (Figure 2 d). Interestingly, the elaborated 5T‐15CN/BVNS heterojunction exhibits superior visible‐light photocatalytic performance for CO_2_ conversion to those of other reported BiVO_4_‐based Z‐Scheme heterojunctions (Table S1).

While substituting the T by SnO_2_ on 15CN/BVNS, the photocatalytic activity for CO_2_ reduction was also largely improved. We also performed the CO_2_ reduction reaction by replacing BVNS with other oxidation half‐reaction photocatalysts such as Fe_2_O_3_ and WO_3_ and observed significant photocatalytic activities for 15CN/Fe_2_O_3_ and 15CN/WO_3_ after loading a certain amount of T (Figure S5). It suggests that such a smart design is also applicable for other narrow‐band gap‐oxides‐based Z‐Scheme heterojunctions. In addition, the photocatalytic overall water splitting (OWS) was also performed with Pt as a co‐catalyst. No H_2_ and O_2_ evolutions were observed for BVNS, CN, or 5T/BVNS as shown in Figure 2 e and Figure 2 f, which is consistent with the earlier reports.[^28^, ^29^] Surprisingly, one can see that both H_2_ and O_2_ are produced with an almost stoichiometric ratio of 2:1 on 15CN/BVNS under UV‐visible‐light irradiation, and the activity is further improved 3‐fold on 5T‐15CN/BVNS. Similar results were obtained under visible‐light irradiation as well (Figure 2 f), however, the optimized 5T‐15CN/BVNS exhibits ≈4‐fold better activity compared to 15CN/BVNS. All these indicate that water is oxidized to produce O_2_ gas when either reduction of CO_2_ or reduction of protons.

Following this novel photocatalytic activity of these junctions, we characterized these materials in detail. As shown in Figure S6 and Figure S7, the characteristic peaks are well assigned to the monoclinic BiVO_4_ (JCPDS no.14‐0688). No noticeable peaks for CN and T are identified in the patterns of *x*CN/BVNS and *y*T‐15CN/BVNS due to the ultrathin characters and high dispersion (*x*=10–20 wt. % and *y*=3–7 wt. %). UV‐Vis diffuse reflectance spectra (DRS) reveal that the absorption edge of BVNS has a blue shift after coupling with CN, which results from the inherent absorption band of CN, whereas no obvious change is observed after further introducing T because of the small loading amounts (Figure S8 and Figure S9).

**Figure 3:**
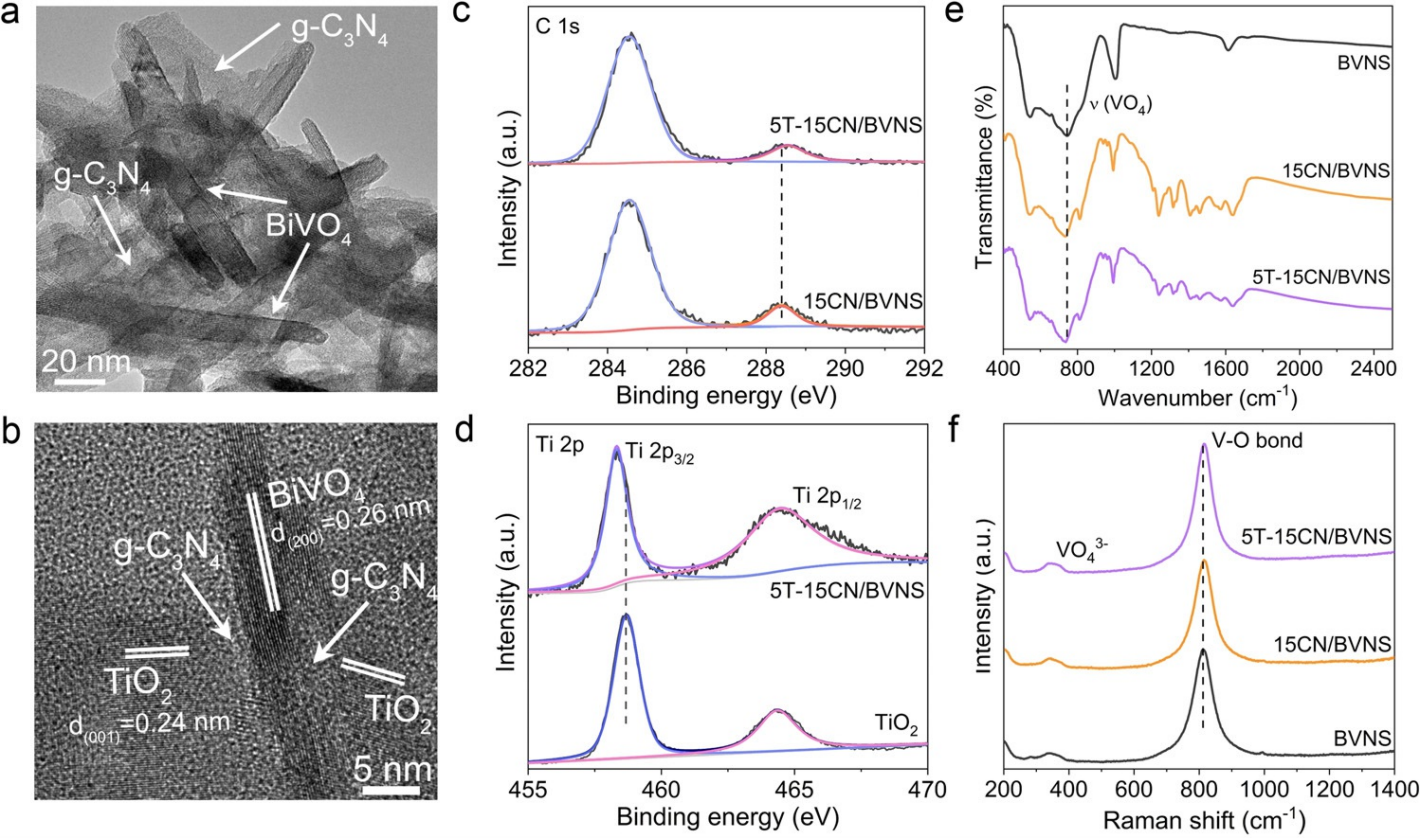
a) TEM image of the resulting 15CN/BVNS heterojunction. b) HRTEM image of 5T‐15CN/BVNS. c) XPS spectra of C 1s of 15CN/BVNS and 5T‐15CN/BVNS. d) XPS spectra of Ti 2p of T and 5T‐15CN/BVNS. e) FTIR spectra and f) Raman spectra of BVNS, 15CN/BVNS, and 5T‐15CN/BVNS.

The transmission electron microscopy (TEM) image shows the sheet‐like morphology for BVNS with a width of 10–30 nm and a length of 80–100 nm (Figure S10). As for 15CN/BVNS, the sheet‐like CN is attached on the BVNS (Figure 3 a). For T introduced samples, 5T‐15CN/BVNS as a representative (Figure 3 b and Figure S11), the sheet‐like T is closely attached to the surface of CN. The HRTEM image clearly shows the intimate ternary heterogeneous interface of 5T‐15CN/BVNS, in which the lattice fringes with the interplanar spacing of 0.26 nm and 0.24 nm are ascribed to (200) plane of BVNS and (001) plane of T, respectively.[^30^, ^31^] The intimate contact structure of 5T‐15CN/BVNS is further tested by energy‐dispersive X‐ray (EDX) elemental mapping (Figure S12) and observed the uniform distribution of Bi, V, O, C, N, and Ti elements across the nanosheet structure. As evidenced by X‐ray photoelectron spectroscopy (XPS), the binding energies of Bi 4*f* and V 2*p* in 15CN/BVNS have a negative shift in comparison with those of BVNS (Figure S13), while the XPS signals of C1*s* and N1*s* shift to the higher binding energies after the introduction of CN (Figure S14). This is due to the Fermi level equilibrium between CN and BVNS when coming into intimate contact. When T is subsequently loaded on 15CN/BVNS to form 5T‐15CN/BVNS, the binding energy of C1*s* displays a slight positive shift compared to 15CN/BVNS (Figure 3 c). On the other hand, the binding energy of Ti 2*p* exhibits a little negative shift compared to bare T (Figure 3 d). The normalized FT‐IR spectra show that the surface hydroxyl groups of CN are significantly increased after HNO_3_ treatment (Figure S15), thus being beneficial to the interfacial interaction. In addition, the characteristic V‐O vibration of BVNS shows a slight blue shift from 745 to 731 cm^−1^ (Figure 3 e). Accordingly, it is suggested that the BVNS and CN be linked via the formed V‐O‐C bonds through hydroxyl group dehydration of the V−O bonds on BVNS and those of C atoms on CN. Meanwhile, T and CN binding was confirmed by C‐O‐Ti bonds resulting from the hydroxyl group dehydration. The Raman spectra of the 15CN/BVNS (Figure 3 f) show, a slight Raman shift for the V−O bond, compared to the BVNS, due to the formation of the V‐O‐C bond. However, the V−O bond signal remains unchanged after loading with T, implying the introduced T is mainly connected with CN rather than BVNS, further supporting the XPS results. All these findings unambiguously indicate that the heterojunction has been successfully constructed, and the well‐designed structure is favorable for the consecutive charge transfer and separation.

**Figure 4:**
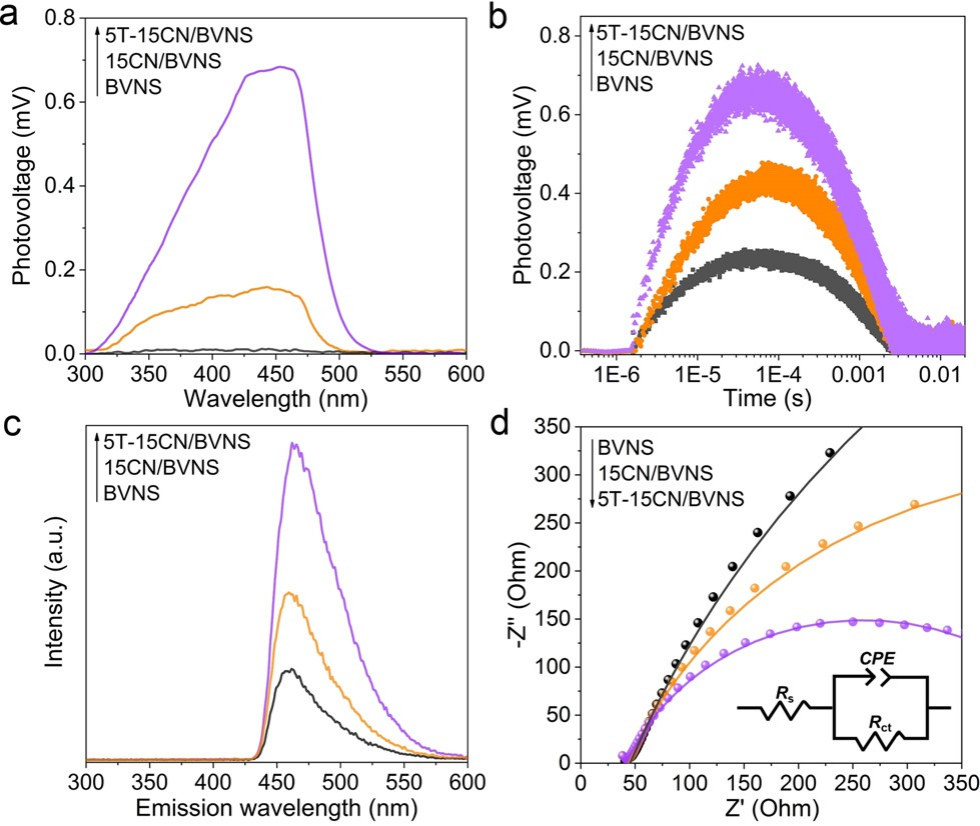
a) SS‐SPS responses in N_2_ atmosphere and b) TPV responses of BVNS, 15CN/BVNS, and 5T‐15CN/BVNS. c) Fluorescence spectra related to the amount of hydroxyl radical formed and d) EIS curves of BVNS, 15CN/BVNS, and 5T‐15CN/BVNS; the insert shows the equivalent circuit model for fitting Nyquist plots.

Steady‐state surface photovoltage spectroscopy (SS‐SPS) is an advanced photophysical technique, which could reveal the photogenerated charge separation and recombination by means of the surface potential difference of a semiconductor before and after illumination. As shown in Figure 4 a and Figure S16, the negligible SPS responses were observed on pristine BVNS under N_2_ atmosphere due to the rapid charge carrier recombination. However, 15CN/BVNS nanocomposite exhibits an obvious SPS signal, indicating the as‐fabricated heterojunction favors charge separation. The best SPS response was observed on 5T‐15CN/BVNS, implying the accelerative charge transfer after the introduction of T. Meanwhile, a stronger transient‐state surface photovoltage (TPV) response is observed on 15CN/BVNS compared to pristine BVNS, and it is much obvious on 5T‐15CN/BVNS, indicating that the charge carrier lifetime is greatly prolonged after introducing T (Figure 4 b).

The charge separation in the fabricated heterojunctions was also analyzed by measuring hydroxyl radicals formed, which are quantified by adding the probe molecule coumarin to generate the luminescent 7‐hydroxy coumarin molecules. The high fluorescence intensity means more hydroxyl radicals are generated, indicating the better charge separation. As shown in Figure 4 c and Figure S17, the highest fluorescence intensity is observed on 5T‐15CN/BVNS heterojunction, confirming the best charge separation. This is consistent with the SPS results. Moreover, as evidenced by the electrochemical impedance spectra (EIS), the radii of semicircle for 5T‐15CN/BVNS decreases remarkably compared to 15CN/BVNS and BVNS, indicating the smallest interfacial resistance (Figure 4 d). The Nyquist plots were fitted to an equivalent circuit (inset in Figure 4 d and Table S2). From the decreasing R_ct_ value of BVNS, 15CN/BVNS, and 5T‐15CN/BVNS, it can be deduced that the introduction of T promotes the charge transfer and separation. As depicted in Figure S18, Figure S19 and Table S3, the optimized one (5T‐15CN/BVNS) exhibits ≈3‐time higher surface charge transfer efficiency (*η*_trans_) compared to 15CN/BVNS.

**Figure 5:**
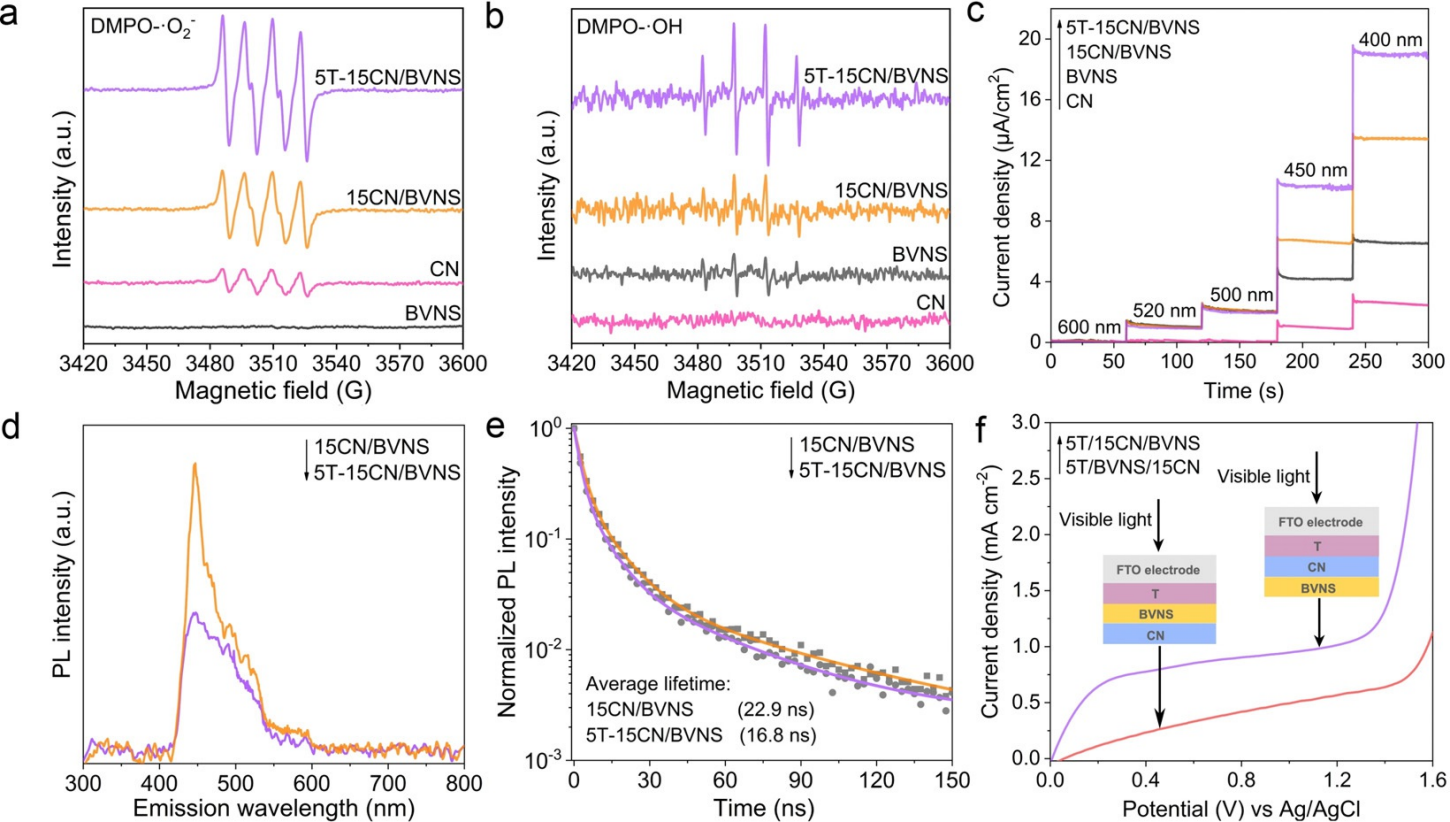
DMPO spin‐trapping EPR spectra recorded for a) ^.^O_2_^−^ and b) ^.^OH under visible‐light irradiation for BVNS, CN, 15CN/BVNS, and 5T‐15CN/BVNS. Detection conditions: the concentration of DMPO is 50 mmol L^−1^. ^.^OH and ^.^O_2_^−^ were determined in aqueous phase and methanolic solution, respectively. c) Photocurrent action spectra of CN, BVNS, 15CN/BVNS, and 5T‐15CN/BVNS under different monochromatic light irradiation. d) PL spectra and e) time‐resolved PL spectra with the excitation wavelength of 405 nm of 15CN/BVNS and 5T‐15CN/BVNS. f) Photoelectrochemical *I*–*V* curves of 5T/15CN/BVNS and 5T/BVNS/15CN with the schematic of the prepared electrodes as the insert. The PEC experiments were carried out in 0.2 M Na_2_SO_4_ electrolyte and the samples were illuminated from the FTO electrode side.

As revealed by Mott‐Schottky curves (Figure S20 and Figure S21), the CB edges of BVNS and CN are determined to be −0.05 V and −1.15 V vs. NHE, respectively. Based on the band gap values estimated by the DRS spectra for BVNS (2.4 eV) and CN (2.7 eV), the valance band positions of BVNS and CN can be located close to 2.35 V and 1.55 V vs. NHE, respectively. The deep VB potential of BVNS and more negative CB potential of CN is highly appropriate to build efficient Z‐Scheme charge transfer between these two materials (Figure S22). The electron paramagnetic resonance (EPR) spectroscopy was further applied to confirm the direction of electrons transfer in the Z‐Scheme system. The strong EPR signals of DMPO‐^.^O_2_^−^ adduct are detected on the CN and heterojunctions, whereas no obvious signal is observed for BVNS, consistent with the thermodynamic requirement that the CB potential of BVNS is more positive than the standard potential of O_2_/^.^O_2_^−^ (−0.33 V vs. NHE). The signal observed for 15CN/BVNS is much higher than pristine CN (Figure 5 a), indicating a successful Z‐Scheme charge transfer between BVNS and CN rather than the type‐II charge transfer (electron transfer from CN to BVNS), therefore more photoelectrons remained on CN for reduction of O_2_. Significantly, for 5T‐15CN/BVNS heterojunction, the intensities of DMPO‐^.^O_2_^−^ characteristic peaks are much stronger than that of 15CN/BVNS, which gives solid proof that the separated electrons further transfer to T and then induce more ^.^O_2_^−^ active species. Meanwhile, the obvious EPR signals assigned to DMPO‐^.^OH are observed on the BVNS and heterojunctions, while no signal is observed on CN because the VB potential of CN is more negative than the potential of H_2_O/^.^OH (1.99 V vs. NHE). As expected, the signal intensities of 15CN/BVNS are stronger than BVNS, and the intensities of DMPO‐^.^OH characteristic peaks are significantly improved after coupling T with 15CN/BVNS heterojunction (Figure 5 b). This unambiguously confirms that the introduced T greatly strengthens the Z‐Scheme charge transfer and separation.

Moreover, the monochromatic photocurrent action spectra were recorded (Figure 5 c). The photocurrent density of BVNS gradually increases as the excitation wavelength decreases from 520 to 400 nm. For CN, it evenly increases from 450 to 400 nm, consistent with its specific absorption. The photocurrent density of 15CN/BVNS obeys the similar law with pure BVNS and CN on the corresponding excitation wavelengths. Noticeably, when both BVNS and CN are excited simultaneously, the recorded photocurrent density sharply becomes large, giving strong evidence for the Z‐Scheme charge transfer in the resulting heterojunction. Interestingly, the 5T‐15CN/BVNS delivers the strongest photocurrent response, accounting for the electron transfer from CN to T. These results are further corroborated by the single wavelength fluorescence spectra (Figure S23).

To gain insights into the promoted charge separation after loading T on 15CN/BVNS, the photoluminescence (PL) spectra from CN with an excitation wavelength of 420 nm were measured (Figure 5 d). The PL intensity of 5T‐15CN/BVNS is quenched compared to 15CN/BVNS, implying the coupled T could effectively inhibit the charge carrier's recombination. The time‐resolved PL spectra (Figure 5 e) further proves this point. The average PL lifetimes of 15CN/BVNS and 5T‐15CN/BVNS with the excitation wavelength of 405 nm are calculated to be 22.9 and 16.8 ns, respectively, suggesting the introduced T provides an additional rapid charge transfer channel for CN. While exciting the 5T‐15CN/BVNS under 355 nm, less PL life decay was observed (Figure S24). This is perhaps due to the accumulation of photogenerated electrons at the CB of the T as UV light excites the T, which is unfavorable for the charge injection from CN to T. The less increase in photocatalytic activities observed under UV‐vis light irradiation compared to visible light can also be explained by the above fact.

To explore the direction of charge transfer, the EPR spectra were measured. It is challenging to monitor the changes of Ti species on the 5T‐15CN/BVNS heterojunction due to the tiny amount of coupled T and the close g values of V^4+^ (*g*=1.96) and Ti^3+^ species (*g*=1.98).[^32^, ^33^] Hence, the EPR spectra of the T/CN sample were carried out at 98 K (Figure S25).The signal with g value of 2.01 is assigned to free electrons from CN,^[34]^ which becomes stronger under visible‐light irradiation. Noticeably, a significant EPR signal for Ti^3+^ species appears under visible‐light irradiation, while no signal observed in dark, suggesting that the photogenerated electrons transferred from CN to T. This result validates our conclusion that the inclusion of T with CN accelerates the Z‐Scheme charge transfer.

In order to further confirm the vital role of T for facilitating the Z‐Scheme charge transfer and separation, three components with diverse sequence were coated on the FTO glass and their photoelectrochemical properties were investigated. In a control experiment, T was placed on top of BVNS (5T/BVNS/15CN) instead of CN (5T/15CN/BVNS), and their photocurrents were measured (Figure 5 f). The photocurrent density of 5T/15CN/BVNS is much higher than that of 5T/BVNS/15CN under visible‐light irradiation, suggesting that the Z‐Scheme charge separation could be facilitated only if T tightly attaches on CN. Meanwhile, it is observed that the interfacial charge‐transfer impedance of 5T/15CN/BVNS is smaller than 5T/BVNS/15CN (Figure S26), which indicates the promoted charge transfer and separation when T and CN are adjacent. These results strongly indicate the inclusion of T on CN greatly enhancing the charge separation rather than T on BVNS. In another control experiment, the random assembled 5T&15CN&BVNS heterojunction with the same ratio as 5T‐15CN/BVNS was also fabricated. The charge separation and photocatalytic activity for CO_2_ reduction observed on 5T&15CN&BVNS are comparable to the 15CN/BVNS, however far less than those of the well‐designed 5T‐15CN/BVNS (Figures S27 and S28). It reveals the electron transfer from CN to T is crucial to facilitate the Z‐Scheme charge transfer and separation. The above experimental results demonstrate that the well‐designed 5T‐15CN/BVNS heterostructures exhibit the exceptional Z‐Scheme charge transfer and separation.

**Figure 6:**
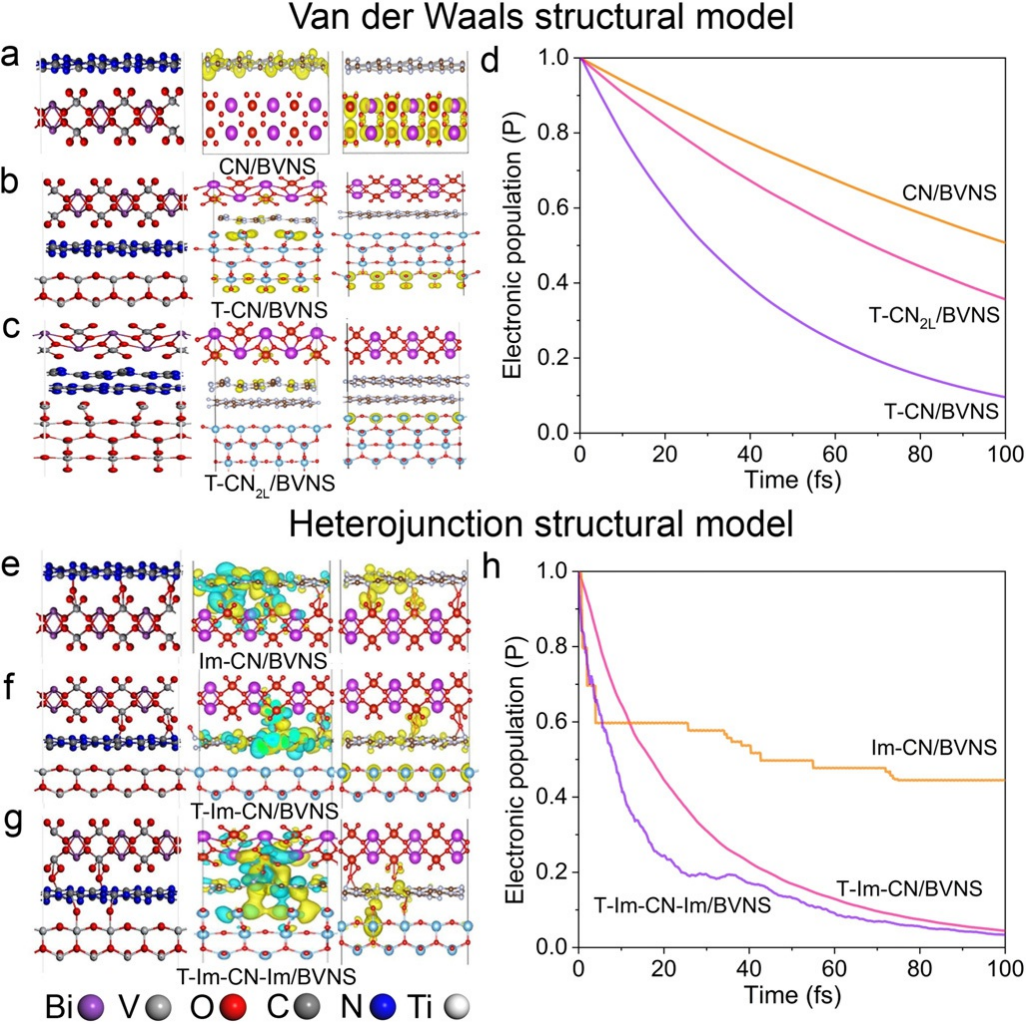
Graphical representations of the interfacial geometries (left panel) and the wave function profiles before (middle panel) and after (right panel) the electron transfer processes of heterostructures a) CN/BVNS, b) T‐CN/BVNS, c) T‐CN_2L_/BVNS. d) The time‐dependent survival probability curves of the excited electrons during the interfacial electron transfer processes based on the nonadiabatic semiclassical quantum dynamics calculation corresponding to heterostructures (a)–(c). e) Hydroxylated interfacial modification corresponding to (a) is also considered as modeling Im‐CN/BVNS. f) One‐side interfacial modified T‐Im‐CN/BVNS. g) Both‐sides interfacial modified T‐Im‐CN‐Im/BVNS. h) The corresponding time‐dependent survival probability curves for the three modified heterostructures (e)–(g). 2 L represents simulated double‐layer CN, and Im‐CN and Im‐CN‐Im represent one‐side interfacial modification and both‐sides interfacial modification, respectively.

The role of the energy platform on Z‐Scheme charge transfer was also clarified by the first‐principles periodic DFT simulations. With the Van der Waals heterojunction as the initial structural model (Figure 6 a–c, Figures S29–S36), it can be found that the CB of CN is the most negative, then T and last the CB of BVNS, indicating the charge transfer pathway in the Z‐Scheme. Based on this model, the charge transfer of CN/BVNS presents a linear characteristic with time, with about 50 % charge transfer after 100 fs. After the introduction of TiO_2_, the charge transfer of ternary heterojunction is significantly promoted, presenting a transfer curve characterized by a quadratic function, and 50 % of the total amount of electrons were transferred within 30 fs (Figure 6 d). While increasing the number of CN layers in the model, the slower charge transfer process was observed compared to the single‐layer model, the holistic charge transfer process could also be facilitated, and 50 % of the total amount of electrons are transferred within 50 fs. To confirm the interfacial interactions of the obtained heterojunction, the hydroxyl group was introduced to form a V‐O‐C bond at the interface of CN and BVNS and other characteristics of heterogeneous junction kept constant. Stable Im‐CN/BVNS and T‐Im‐CN/BVNS systems (Im‐CN/BVNS represents the hydroxylated CN/BVNS) are obtained by optimizing the V‐O‐C structure (Figure 6 e–h, Figures S37–S40). The V‐O‐C bonds in the Im‐CN/BVNS system promote the rapid charge transfer of the excited electron within 10 fs, however, later the charge transfer characteristics are gradually weakened, which is concussive and slow within 100 fs. This torpid process may be relative to the competitive charge transfer dominated by the type II heterojunction. The V‐O‐C bond in T‐Im‐CN/BVNS also has the same effect, however, due to the presence of TiO_2_, the charge transfer has a more obvious quadratic function characteristic. Compared with Van der Waals T‐CN/BVNS model, the T‐Im‐CN/BVNS model is more encouraged to study the charge transfer. A 50 % of the charge transfer can be completed within 20 fs on T‐Im‐CN/BVNS although the charge transfer is not as fast as Im‐CN/BVNS (10 fs). This is mainly due to the modified CN and the subsequently introduced TiO_2_ slightly upshifting the energy band position of the system. Besides, the impact of possible Ti‐O‐C bonding is also considered. When the V‐O‐C structure is optimized, the Ti‐O‐C bond can be constructed nearby, whereafter, the overall T‐Im‐CN‐Im/BVNS (T‐Im‐CN‐Im/BVNS represents hydroxylated interfacial modified T, CN, and BVNS) can achieve metastable structure. The charge transfer feature also well preserves the more rapid charge transfer within 10 fs, and 80 % transfer occurs within 20 fs, surprisingly. These theoretical results indicate that the Z‐Scheme charge transfer is greatly facilitated by introducing T and supporting our experimental results.

To explore the photocatalytic reaction mechanism, electrochemical experiments were firstly performed in different gas‐bubbled systems (Figure S41). The onset overpotentials of BVNS and 15CN/BVNS are comparable in the N_2_‐bubbled system, while it is much lower for 5T‐15CN/BVNS, well demonstrating that the coupled T is advantageous for H_2_O activation towards H_2_ evolution. This is in good agreement with the earlier report that the CB position of T is thermodynamically available for H_2_ production.^[10]^ Similarly, the reduction onset potentials were measured in the CO_2_‐bubbled system and found that the 5T‐15CN/BVNS shows the equivalent onset potential to the N_2_‐bubbled system. It suggests that the introduced T is more favorable for H_2_O activation to produce H atoms first and then initiate the CO_2_ reduction reaction. To further confirm the role of protons in the subsequent conversion of CO_2_, isotopic D_2_O experiments were employed under identical photocatalytic reaction conditions. A certain amount of CH_3_OH was deliberately added in the reaction system as a hole scavenger in order to investigate the behavior of the photoelectrons. The photocatalytic products were analyzed by GC‐MS method. As shown in Figure S42, when H_2_O was substituted with D_2_O, the CD_4_ and the related fragments were detected, suggesting that the electrons activated D_2_O to produce D atom and then initiated the reduction of CO_2_ to form CD_4_.

The charge separation of 15CN/BVNS could also be substantially enhanced by replacing the T with SnO_2_ (Figure S43). Likewise replacing the BVNS by other narrow‐band gap‐oxides (e.g., Fe_2_O_3_ and WO_3_, see Figure S44 and Figure S45 for structural characterizations) in the present Z‐Scheme heterojunctions, similar results on CO_2_ photoreduction were obtained (Figure S5). For instance, after integrating a certain amount of T, the SPS responses of 15CN/Fe_2_O_3_ and 15CN/WO_3_ are greatly enhanced, which undoubtedly evidences the versatility of the proposed energy‐platform strategy (Figure S46 and Figure S47).

**Figure 7:**
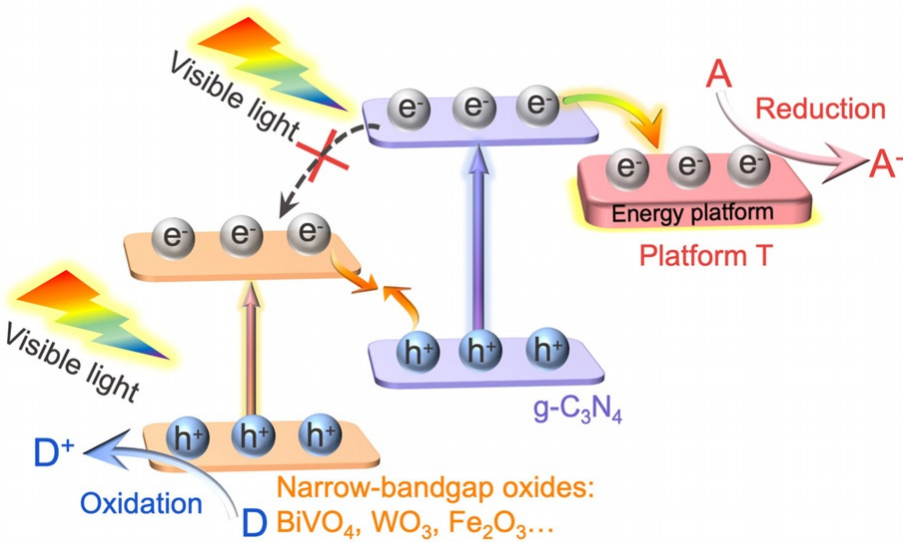
Schematic representation of the proposed cascade Z‐Scheme mechanism of photogenerated charge transfer under visible light for efficient photocatalysis. T refers to (001)TiO_2_, which can feasibly be replaced by other wide bandgap semiconductors like SnO_2_.

Accordingly, the mechanism of cascade Z‐Scheme charge transfer and separation is proposed (Figure 7). Under visible‐light irradiation, the photoelectrons in the CB of BVNS recombine with the holes in the VB of CN, and the powerful holes left in the VB of BVNS induce the water oxidation to produce O_2_. The photogenerated electrons from the CB of CN are transferred to the introduced T to initiate the CO_2_ reduction reaction. It is worth noting that the Z‐Scheme charge transfer and separation is accelerated and maximized by fabricating the dimension‐matched constituents through their tightly connected interface. Most importantly, the photogenerated electrons of CN could be timely transferred to the introduced additional energy platform, to inhibit the electron accumulation in the CB of CN, greatly prolonging the lifetime of photogenerated electrons. Thus, the present cascade Z‐Scheme charge transfer system is successfully demonstrated for efficient artificial photocatalysis.

## Conclusion

In summary, a universal strategy has been demonstrated to fabricate a cascade Z‐Scheme heterojunction, in which an effective energy platform is crucial to direct the Z‐Scheme charge transfer and separation for efficient photocatalysis without a significant loss of both reduction and oxidation potentials. The dimension‐matched 5T‐15CN/BVNS heterojunction exhibits nearly 20 times better performance for CO_2_ reduction by water in the absence of any cocatalysts and costly sacrificial agents compared with the advanced BiVO_4_ nanosheet photocatalyst, even superior to other Z‐Scheme systems with noble metal as mediators. Meanwhile, the photocatalytic activity for overall water splitting over 5T‐15CN/BVNS also exhibits ≈4‐fold improvement compared with 15CN/BVNS under visible light. All these are believed due to the directed charge transfer between CN and BVNS promoted by TiO_2_ as the electron‐energy platform, which has been strongly supported by both comprehensive spectroscopic measurements and theoretical simulation. Importantly, this strategy is also suitable to facilitate the charge transfer in other Z‐Scheme heterojunctions (eg.C_3_N_4_/WO_3_ and C_3_N_4_/Fe_2_O_3_), and other wide‐band gap semiconductors, such as SnO_2_, can also be used as an alternative electron‐energy platform. This work opens up new avenues for the rational design of a cascade Z‐Scheme charge transfer system for efficient solar‐to‐fuel conversion.

## Conflict of interest

The authors declare no conflict of interest.

## Supporting information

As a service to our authors and readers, this journal provides supporting information supplied by the authors. Such materials are peer reviewed and may be re‐organized for online delivery, but are not copy‐edited or typeset. Technical support issues arising from supporting information (other than missing files) should be addressed to the authors.

Supporting Information
