# Supplementary material for: Energy Platform for Directed Charge Transfer in the Cascade Z‐Scheme Heterojunction: CO2 Photoreduction without a Cocatalyst
Source: Angew Chem Int Ed Engl. 2021 Aug 13;60(38):20906–14. doi: 10.1002/anie.202106929 (PMC8518548; doi:10.1002/anie.202106929)
Supplement: Supplementary file 1 — Supporting Information [file ANIE-60-20906-s001.pdf]

Angewandte Chemie
Eine Zeitschrift der Gesellschaft Deutscher Chemiker

Supporting Information

Energy Platform for Directed Charge Transfer in the Cascade Z-Scheme Heterojunction: CO_2 Photoreduction without a Cocatalyst

Ji Bian[+], Ziqing Zhang[+], Jiannan Feng, Madasamy Thangamuthu, Fan Yang, Ling Sun, Zhijun Li, Yang Qu, Dongyan Tang, Zewei Lin, Fuquan Bai, Junwang Tang,* and Liqiang Jing**

anie_202106929_sm_miscellaneous_information.pdf

Experimental section

Synthesis of $BiVO_4$ nanosheets. In a typical synthesis, 2.21 g of $BiCl_3$ and 1.05 g of cetyltrimethylammonium bromide (CTAB) were dissolved in 60 mL of ethylene glycol under vigorous magnetic stirring. Then, 2.80 g of $NaVO_3$ was added into the above solution followed by stirring for 30 min. Then, the mixture was transferred into a 50 mL Teflon-lined autoclave, sealed, and heated at 120ºC for 12 h. After the system was cooled down to room temperature, the resulting products were washed with ethanol and deionized water several times, and then dried at 60ºC in a vacuum oven. Finally, the obtained sample was fast calcined for 8 min at 400ºC and represented as BVNS.

Synthesis of hydroxylated C_3N_4 nanosheets. Hydroxylated C_3N_4 nanosheet was prepared by a modified method based on a previous report.[1] In a typical synthesis, 1 g of melamine and 1.2 g of phosphorous acid were dissolved in 100 mL of deionized water under vigorous magnetic stirring at 80ºC in a water bath. Then the mixture was transferred into a 100 mL Teflon-lined autoclave, sealed, and heated at 180ºC for 10 h. The resulting products were washed with deionized water several times and dried at 60ºC in an oven. Afterward, 0.6 g of the prepared powder was refluxed with a mixed aqueous solution of 5 ml glycerol and 15 ml ethanol for 3 h at 90ºC. Then, the obtained products were washed by ethanol several times and dried at 60ºC. Subsequently, the resultant solids were heated to 500ºC in a muffle furnace with a heating rate of 2ºC/min for 2 hours. To obtain the hydroxylated C_3N_4 nanosheet, the above few-layers C_3N_4 were immersed in HNO_3 solution and refluxed boiling at 130ºC for 6 h followed by washing with water until neutralizing the solution, which is dried in an oven. This sample is represented as CN.

Synthesis of (001) facet-exposed anatase TiO_2 nanosheets. In a typical procedure, 5 mL of $Ti(OBu)_4$ was mixed with 20 mL of absolute ethanol under vigorous stirring. After that, a hydrofluoric acid solution (0.9 mL, 40%) was added. The resulting solution was stirred for 1 h and transferred to a Teflon lined stainless-steel autoclave, and kept at 160ºC for 24 h. After the system was cooled to room temperature, the white precipitate was collected, washed with ethanol and deionized water several times by turns, and named as T.

Synthesis of CN coupled BVNS. The CN coupled BVNS were prepared by the hydroxyl induced assembly method. In the typical synthesis, a proper amount of CN and BVNS were dispersed in 60 mL of ethanol, and ultrasound for 30 min. Then the above mixture was refluxed at 80ºC for 2 h. Afterward, the obtained samples were washed with deionized water several times and dried at 60ºC in an oven. This sample was represented as xCN/BVNS, where x (10, 15, and 20), which is determined by the mass ratio percentage of CN to BVNS.

Synthesis of (001) facet-exposed TiO_2 nanosheets coupled CN/BVNS heterojunction. The (001) facet-exposed TiO_2 nanosheets coupled CN/BVNS

heterojunction was synthesized through a two-step hydroxyl-induced assembly strategy. First, 15CN/BVNS heterojunction was synthesized as described above, and then the T was assembled on it as follows. A proper amount of T and 15CN/BVNS were dispersed in 60 mL of ethanol and ultrasonicated for 30 min. Then, the above mixture was refluxed at 80ºC for 2 h. The obtained samples were washed with deionized water several times and dried at 60 ºC in an oven. This sample was named as yT-15CN/BVNS, where y (3, 5, and 7), which is determined by the mass ratio percentage of T to BVNS.

Synthesis of α-Fe_2O_3. In a typical procedure, 10 mL of 5 wt% ammonia solution was added into a 50 mL Teflon-lined autoclave. Then, a weighing bottle containing a mixture of 0.8 g $Fe(NO_3)_3 \cdot 9H_2O$ and 8 mL n-butyl alcohol was placed in the autoclave with a support to separate with the ammonia solution. The Teflon-lined autoclave was kept at 140 ºC for 6 h in an oven. After being cooled naturally to the room temperature, the sample was washed for several times with ethanol and deionized water in turn and then dried in an oven at 80 ºC. Finally, the samples were calcined in air at 400ºC for 2h.

Synthesis of CN coupled Fe_2O_3. In the typical synthesis, a proper amount of CN and Fe_2O_3 were dispersed in 60 mL of ethanol, and ultrasound for 30 min. Then the above mixture was refluxed at 80ºC for 2 h. Afterwards, the obtained samples were washed with deionized water for several times, and dried at 60ºC in an oven. This sample was represented as CN/Fe_2O_3.

Synthesis of (001) facet-exposed TiO_2 nanosheets (T) coupled CN/Fe_2O_3 heterojunction. In a typical procedure, the CN/Fe_2O_3 heterojunction was synthesized as described above, and then the T was assembled on it as follows. A proper amount of T and CN/Fe_2O_3 were dispersed in 60 mL of ethanol, and ultrasonicated for 30 min. Then, the above mixture was refluxed at 80ºC for 2 h. Afterwards, the obtained samples were washed with deionized water for several times, and dried at 60ºC in an oven. This sample was represented as T-CN/Fe_2O_3.

Synthesis of WO_3. In a typical procedure, 0.22 g of polyethylene oxide-polypropylene oxide-polyethylen (P123) (Pluronic, M=5800) was dissolved in 14.44 g of ethanol absolute under vigorous magnetic stirring. Then, a certain amount of deionized water and 1.5 mL of ethylene glycol was added to the above mixture and stirred for 2 h to form a clear solution. Finally, the solution was sealed and kept in a brown reagent bottle at least 48 h before using. The solution was named as solution P. 0.44 g of WCl_6 was added to 16.83 g of solution P, and stirred for 20 min to obtain a yellow solution. The solution was then transferred to a 50 mL of Teflon-lined autoclave to be kept under 110°C for 3 h, and naturally cooled to room temperature. After the solvothermal treatment, the precipitate was washed with absolute ethanol, then dried in a vacuum oven at 80°C for 12 h. After that, the obtained sample was calcined for 1 h at 400ºC and represented as WO_3.

Synthesis of CN coupled WO_3. In the typical synthesis, a proper amount of CN and WO_3 were dispersed in 60 mL of ethanol, and ultrasound for 30 min. Then the above mixture was refluxed at 80ºC for 2 h. Afterwards, the obtained samples were washed with deionized water for several times, and dried at 60ºC in an oven. This sample was represented as CN/WO_3.

Synthesis of (001) facet-exposed TiO_2 nanosheets (T) coupled CN/WO_3 heterojunction. In a typical procedure, the CN/WO_3 heterojunction was synthesized as described above, and then the T was assembled on it as follows. A proper amount of T and CN/WO_3 were dispersed in 60 mL of ethanol, and ultrasonicated for 30 min. Then, the above mixture was refluxed at 80ºC for 2 h. Afterwards, the obtained samples were washed with deionized water for several times, and dried at 60ºC in an oven. This sample was represented as T-CN/WO_3.

Synthesis of SnO_2 nanoparticles. In the typical synthesis, 2 g of $SnCl_4 \cdot 5H_2O$ was dissolved in deionized water and NaOH solution was added dropwise to it under continuous stirring. At the beginning, a white cloudy suspension was formed in the acidic pH range which slowly disappeared with the addition of more NaOH solution. After the pH value reached about 12, solution became transparent. This solution was transferred to 100 mL Teflon-lined stainless steel autoclave and calcined at 200°C for 12 h. The product was cooled to room temperature naturally and washed three times with distilled water to remove impurities. The purified white powder was dried in an oven overnight at 60°C to obtain SnO_2 nanoparticles.

Synthesis of SnO_2 coupled CN/BVNS heterojunction. In a typical procedure, the 15CN/BVNS heterojunction was synthesized as described before, and then the SnO_2 was assembled on it as follows. A proper amount of SnO_2 and 15CN/BVNS were dispersed in 60 mL of ethanol and ultrasonicated for 30 min. Then, the above mixture was refluxed at 80ºC for 2 h. The obtained samples were washed with deionized water several times and dried at 60ºC in an oven. This sample was named as SnO_2-15CN/BVNS.

Preparation of 5T/BVNS/15CN and 5T/15CN/BVNS electrodes. Both 5T/BVNS/15CN and 5T/15CN/BVNS were obtained by blade-coating the dispersion of these components on FTO glass in sequence, followed by calcination at 300 °C for 30 min in air. In a typical procedure, for 5T/BVNS/15CN photoelectrode synthesis, BVNS, CN and T were firstly dispersed in a mixture solution of Nafion and ethanol with the volume ratio of 1:9 under vigorous stirring to make individual solution, respectively. Then a certain amount of T droplets was dropped on a FTO glass substrate, followed by a blade-coating procedure. Then, the CN was dispersed on the T coated FTO glass, and last the BVNS was dispersed on the substrate. Finally, the sample was calcined at 300 °C for 30 min in a muffle furnace, denoted as 5T/15CN/BVNS. Similarly, 5T/BVNS/15CN electrode was obtained by a same method but with a different coating sequence, swapping CN and BVNS.

Characterizations

The X-ray powder diffraction (XRD) patterns of the samples were collected by a Bruker D8 advance diffractometer with CuKα radiation. The UV-Vis diffuse reflectance spectra (UV-Vis DRS) of the samples were acquired using Shimadzu UV 2550 spectrophotometer with $BaSO_4$ as a reference. The Fourier-transform infrared (FT-IR) spectra of the samples were recorded with a Bruker Equiox 55 spectrometer, KBr as the diluents. The morphologies of the samples were analyzed by transmission electron microscopy (TEM) on an FEI Tecnai G2 S-Twin instrument with an acceleration voltage of 200 kV. The FEI Tecnai G2 S-Twin equipped with Energy-dispersive X-ray (EDX) Detector was used to acquire element analysis of the samples. The surface photovoltage spectroscopy (SPS) measurements were carried on home-built apparatus, equipped with a lock-in amplifier (SR830, USA) synchronized with a light chopper (SR540, USA). The transient-state surface photovoltage (TPV) responses of the samples were recorded in the air at room temperature. The samples were excited by a radiation pulse of 355 nm with 10 ns width from the second harmonic of a neodymium-doped yttrium aluminum garnet (Nd:YAG) laser (Lab-130-10H, Newport, Co.). The signals were amplified with a preamplifier and registered with a 1 GHz digital phosphor oscilloscope (DPO 4104B, Tektronix). The Raman spectra were recorded with a Renishaw inVia Confocal Raman spectrometer with a 785 nm laser as the excitation source. The electron paramagnetic resonance (EPR) measurements were carried out on a Bruker EMX plus model spectrometer operating at the X-band frequency. The reactive $\bullet O_2^-$ and •OH species were detected by using 5,5-dimethyl-1-pyrroline N-oxide (DMPO) as a spin trap under visible-light irradiation. The X-ray photoelectron spectroscopy (XPS) was performed by Kratos-AXIS ULTRA DLD. Al (Mono) was used as the X-ray source.

Hydroxyl radical measurement. Hydroxyl radicals (•OH) are important active species in photocatalytic reaction. Using coumarin as a labeled molecule to detect the content of hydroxyl radicals is an effective method with high sensitivity. The specific method for the hydroxyl radical test is as follows: 0.02 g of the catalyst was placed in 50 mL of coumarin solution at a concentration of 2×10^{-4} M. The mixture was stirred for 30 min before the experiment, to ensure that it reached the adsorption-desorption equilibrium. After 1 h irradiation, appropriate amount of the suspension was centrifuged and the supernatant was transferred into a Pyrex glass cell for the fluorescence measurement of 7-hydroxycoumarin by a spectrofluorometer (Perkin-Elmer LS55). To cut off UV-light, a light filter of 420 nm was placed between the light source and the reactor.

Photoelectrochemical and electrochemical measurements. Photoelectrochemical (PEC) and electrochemical reduction measurements were carried out in a traditional three-electrode system. The prepared thin film electrode was used as a working electrode, a platinum plate (99.9%) as the counter electrode, and an Ag/AgCl as the reference electrode. A 0.2 M Na_2SO_4 solution as the electrolyte. High purity nitrogen gas (99.999%) was bubbled through the electrolyte before and during the experiments. PEC experiments were performed in a quartz cell using a 500 W xenon lamp with a

cut-off filter (λ > 420 nm) as the illumination source. An IVIUM V13806 electrochemical workstation was employed to test the photoelectrochemical and electrochemical performance of the series of photocatalysts. All the experiments were performed at room temperature (about 25 ºC).

The surface charge transfer efficiency (η_{trans}) was calculated using the following equations:

$$\eta_{trans}=\frac{J_{H_2O}}{J_{Na_2\,SO_3}}$$

J_{H_2O} and $J_{Na_2SO_3}$ are the photocurrent densities obtained in 0.2 M Na_2SO_4 electrolytes (pH 6.8) at the applied potential of 0.2 V *versus* Ag/AgCl without and with 0.1 M Na_2SO_3 as an efficient hole scavenger, respectively.

Photocatalytic activities for CO_2 conversion. 0.1 g of photocatalyst powder was suspended in 5 mL of water with magnetic stirring in a quartz reactor. A 300 W Xenon lamp was used as the light source with a 420 nm cut-off filter. High pure CO_2 gas was passed through water and then entered into the reaction setup for reaching ambient pressure. The photocatalyst was allowed to equilibrate in the CO_2/H_2O system for 20 min and then irradiated for 4 hours. During irradiation, about 0.25 mL of gas produced was taken from the reaction cell at given time intervals for CO and CH_4 concentration analysis using a gas chromatograph (GC-7920 with both TCD and FID detectors, Au Light, Beijing), and for O_2 evolution analysis using a gas chromatograph (GC-7900 with TCD, Perfect Light, Beijing). The isotope-labeled experiment was carried out using $^{13}CO_2$ instead of CO_2, and the reduction products were analyzed on a gas chromatography−mass spectrometry (GC-MS, MS5977A, Agilent). The isotopic D_2O labelled experiment was conducted under identical photocatalytic CO_2 reduction reaction conditions, but H_2O was substituted with a mixture of D_2O and methanol. The reduction products were analyzed on a gas chromatography−mass spectrometry (GC-MS, MS5977A, Agilent).

Photocatalytic activities for Overall water splitting. The overall water splitting test was conducted by an integrated system composed of a photoreactor and an online analytic system (Perfectlight, Beijing, Labsolar-6A circulation system). In detail, 0.1 g of photocatalyst powder was dispersed in 100 mL of deionized water with a calculated amount of H_2PtCl_6 as the cocatalyst precursor in a cubic glass cell and kept stirring. Ahead of the reaction, the mixture was deaerated by evacuation to remove O_2 and CO_2 dissolved in water. Subsequently, the reactor was exposed under a 300 W Xe lamp with a cutoff filter (λ≥420 nm). The amount of the evolved H_2 and O_2 in the photocatalysis was analyzed by an online gas chromatograph (GC-2002, TCD, molecular sieve 5 Å, Ar carrier). The gas tight reactor can be guaranteed by measuring an extremely low leakage rate (≤5×10^{-5} Pa•L/s), and the amount of O_2 leakage for 24 h is less than 1 μmol.

DFT study. All theoretical calculations were performed by the density functional theory (DFT) pseudopotential plane-wave method as implemented in the Vienna Ab initio

simulation package (VASP).[2,3] The all-electron projected argument wave (PAW)[4] potential was used to describe the ion-electron interaction. The exchange and correlation potential were described with the Perdew−Burke−Ernzerhof (PBE)[5] of the generalized gradient approximation (GGA). A van der Waals (vdW) interaction was described by the DFT-D2 method of Grimme.[6] The cutoff energy was set as 520 eV, and structure relaxation was performed until the convergence criteria of energy and force reached 1×10^{-5} eV and 0.02 eV $Å^{-1}$, respectively. The Brillouin zone was sampled with 3×2×1 and 6×4×1 Monkhorst-Pack K-point mesh for the structure optimizations of $BiVO_4$, C_3N_4, and anatase TiO_2 and electronic structure calculations of $BiVO_4/C_3N_4/TiO_2$ heterostructure or with their hydroxylated interface, respectively. And the band structure of the $BiVO_4/C_3N_4/TiO_2$ heterostructure was calculated along the G→X→S→Y→G path. In the DFT-D2 scheme, all force field parameters are obtained based on the PBE functional. The detailed information of the ultrafast interfacial electron transfer (IET) processes is carried out using the semi-classical quantum dynamics method based on semiempirical EH Hamiltonian.[7] The EH method has been widely used to calculate the electronic structure of periodic condensed matter systems. It requires a small number of transferable parameters and can provide a reliable description of the chemical bonding and energy band of both elemental and bulk materials with relatively small computation expense. The IET computational details please refer to the literature for details and the calculation method therein.[8]

The optimized the lattice parameters were a=5.214 Å, b=5.084 Å for unit cell of $BiVO_4$, a=10.886 Å, b=15.104 Å for C_3N_4 monolayer and a=b=3.776 Å, c=9.486 Å for unit cell of anatase TiO_2. These values are in good agreement with experiment values.[9, 10] The heterostructure was constructed by 2×3×1 unit of $BiVO_4$, C_3N_4 and 3×4×1 unit of anatase TiO_2 monolayer. The anatase TiO_2 monolayer was chosen from (001) face and (100) face. The lattice parameters were a= 11.328 Å, b=15.104 Å for (001) TiO_2 and a=9.486 Å, b=15.104 Å for (100) TiO_2. The mismatch defined as $\varepsilon = \left|a_{\mathrm{TiO2}} - a_{\mathrm{BiVO4}}\right| \times 3 / \left|a_{\mathrm{TiO2}} + a_{\mathrm{C3N4}} + a_{\mathrm{BiVO4}}\right|$ for the parameters a and b of the heterostructure containing (001) TiO_2 is only 4.9% and 0.9% which are in acceptable range. The mismatch for the parameters a and b of the heterostructure containing (100) TiO_2 is 7.4% and 0.9%. A vacuum zone of 20 Å was used to minimize the interactions between the adjacent systems for the $BiVO_4/C_3N_4/TiO_2$ heterostructure. The interlayer distances between three layers are 2.87 Å and 3.01 Å for the containing (100) TiO_2 heterostructures and containing (001) TiO_2 heterostructures, respectively.

Figures

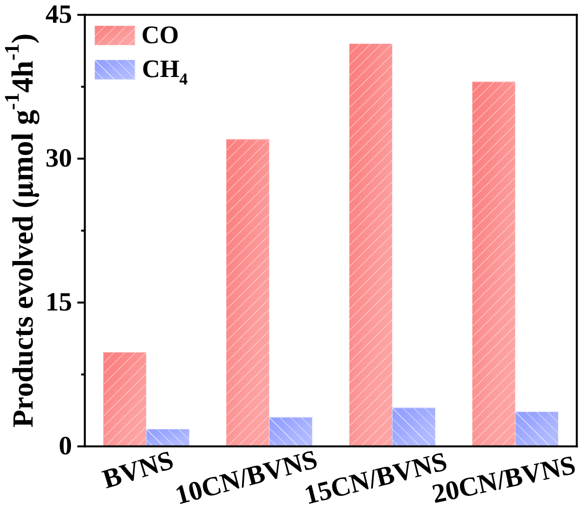

Figure S1 Photocatalytic activities for CO_2 conversion of BVNS and xCN/BVNS under UV-visible-light irradiation for 4 h.

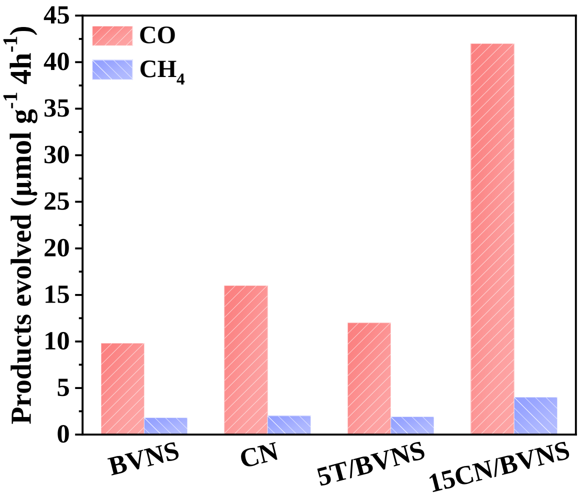

Figure S2 Photocatalytic activities for CO_2 conversion of BVNS, CN, 5T/BVNS and 15CN/BVNS under UV-visible-light irradiation for 4 h.

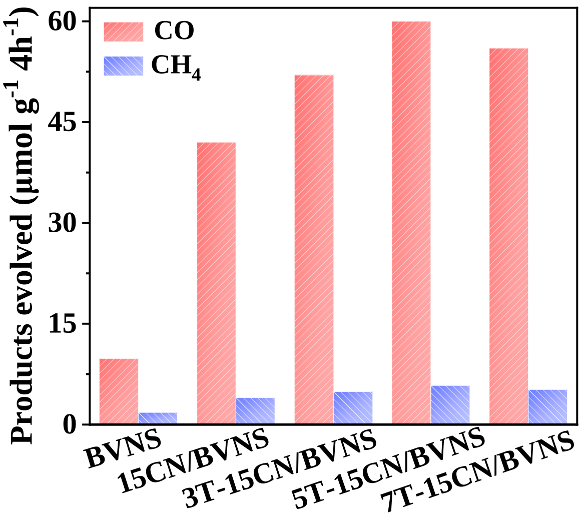

Figure S3 Photocatalytic activities for CO_2 conversion of BVNS, 15CN/BVNS and yT-15CN/BVNS under UV-visible-light irradiation for 4 h.

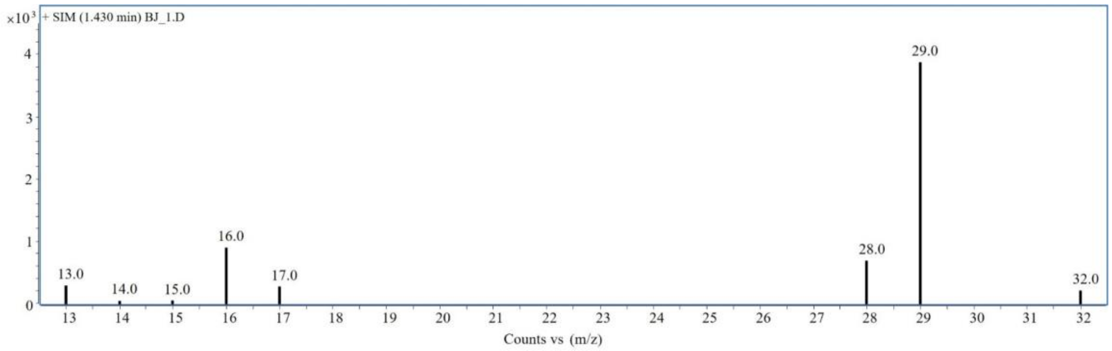

Figure S4 Mass spectroscopy of the products over 5T-15CN/BVNS in photocatalytic reduction of $^{13}CO_2$. The major products ^{13}CO (m/z = 29) and $^{13}CH_4$ (m/z = 17) and their fragments are observed.

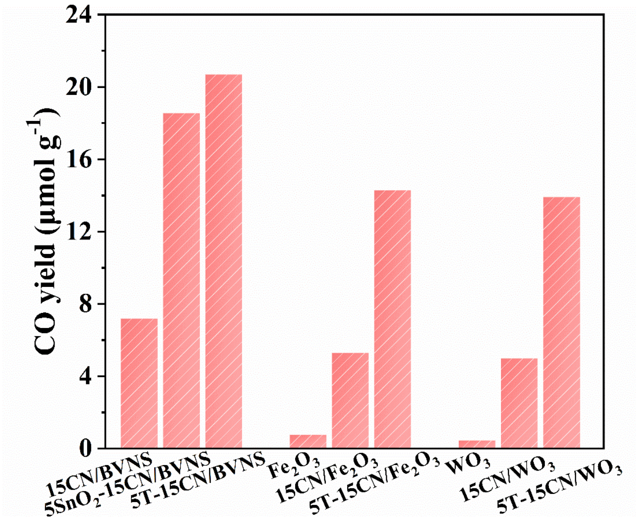

Figure S5 Photocatalytic activities for CO_2 conversion of different samples under visible-light irradiation for 4 h.

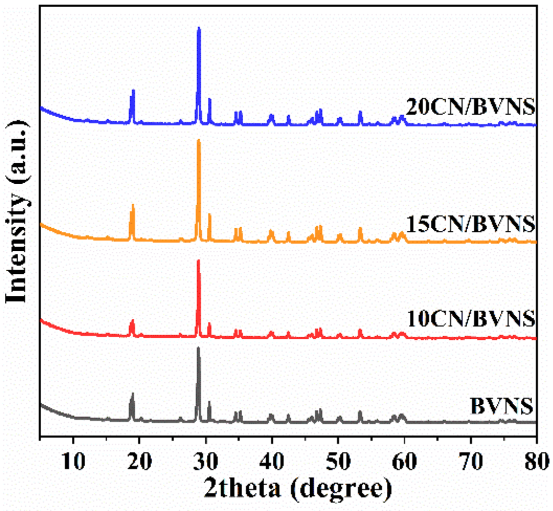

Figure S6 XRD patterns of BVNS and xCN/BVNS nanocomposite.

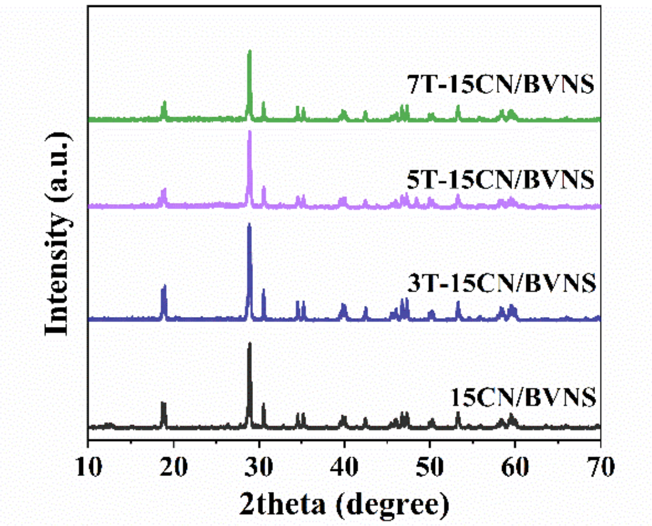

Figure S7 XRD patterns of 15CN/BVNS and yT-15CN/BVNS.

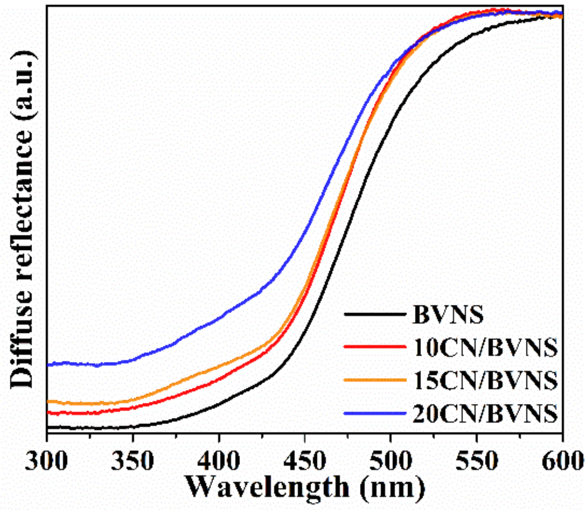

Figure S8 DRS spectra of BVNS and xCN/BVNS nanocomposite.

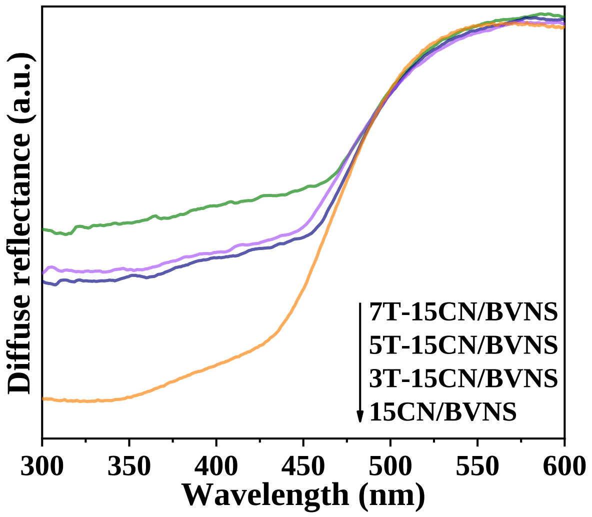

Figure S9 DRS spectra of 15CN/BVNS and yT-15CN/BVNS.

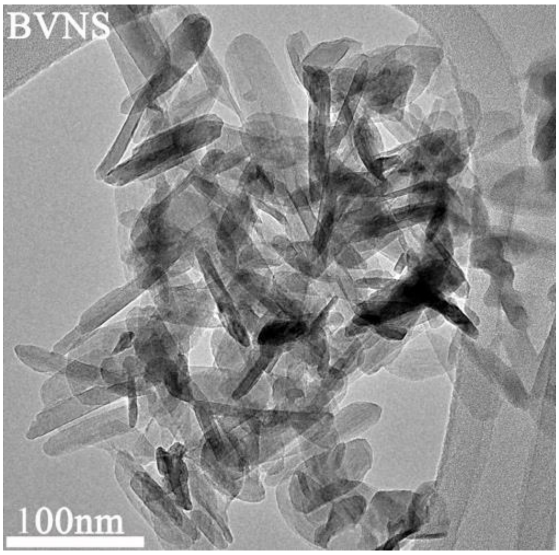

Figure S10 TEM image of BVNS.

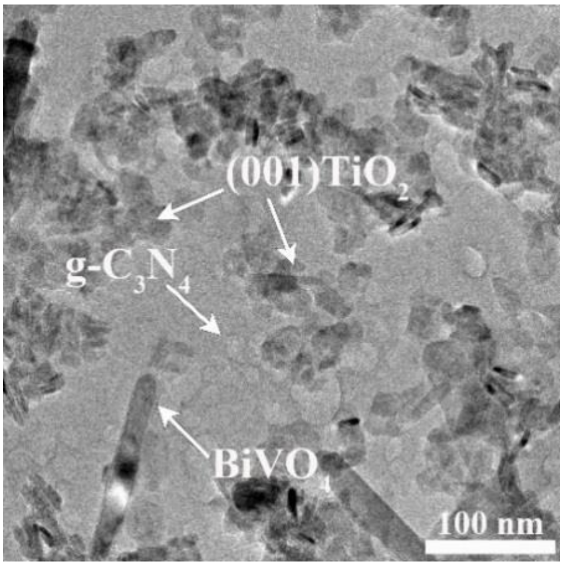

Figure S11 TEM image of 5T-15CN/BVNS.

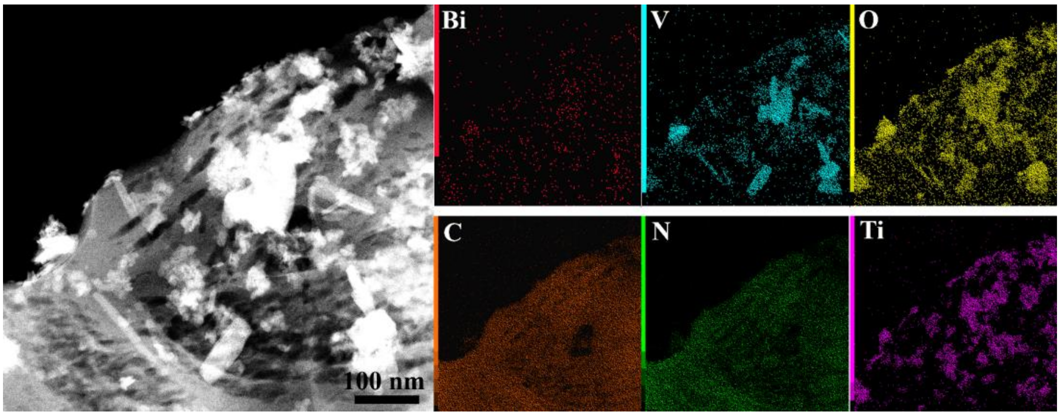

Figure S12 STEM image of 5T-15CN/BVNS and the corresponding EDX mapping images of elemental Bi, V, O, C, N and Ti.

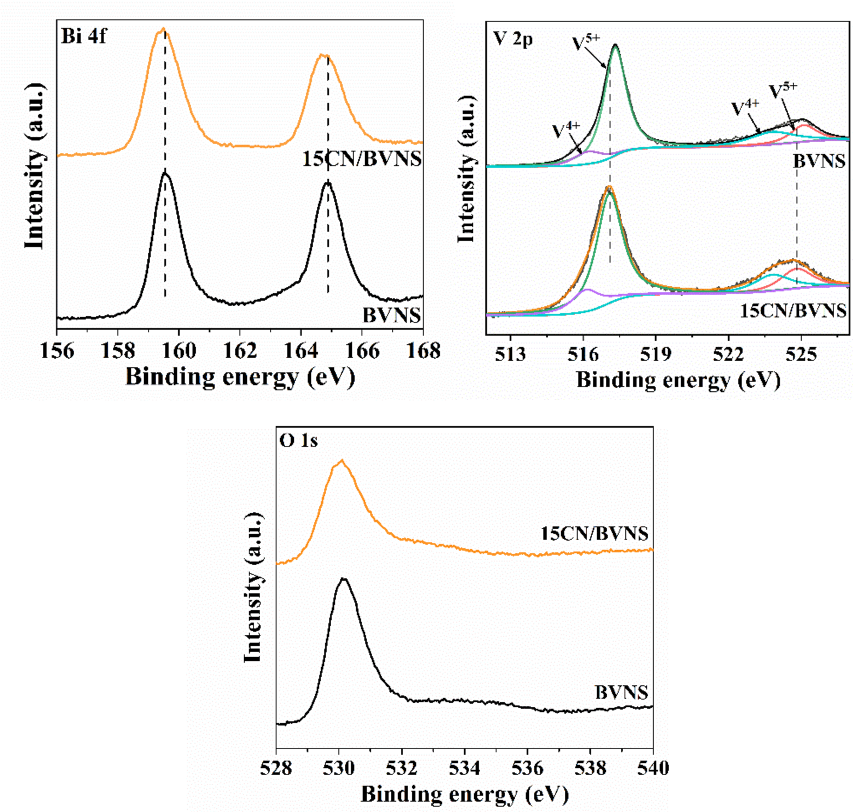

Figure S13 XPS spectra of Bi 4*f*, V 2*p* and O 1*s* of BVNS and 15CN/BVNS.

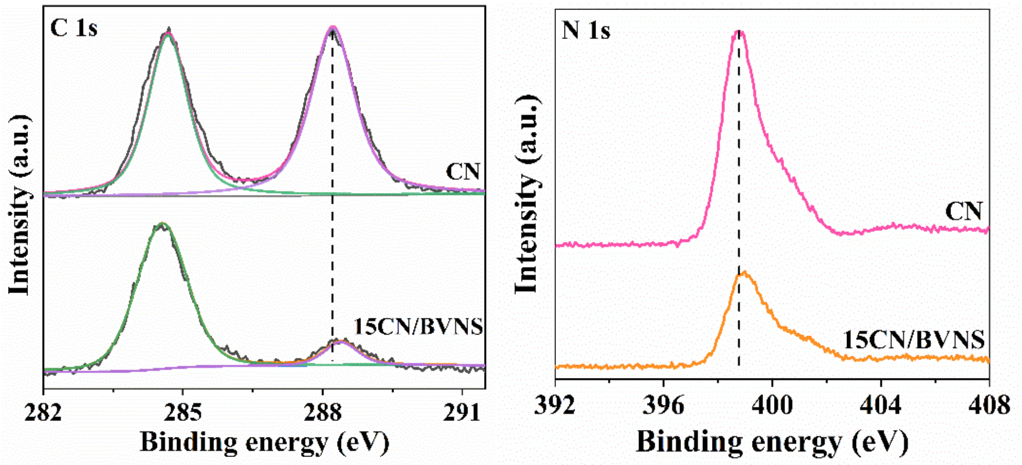

Figure S14 XPS spectra of C 1*s* and N 1*s* of CN and 15CN/BVNS.

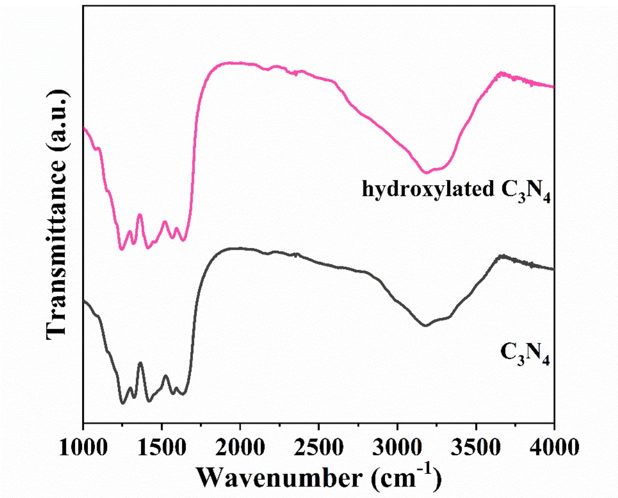

Figure S15 FT-IR spectra of C_3N_4 and HNO_3-treated hydroxylated C_3N_4, where the broad peak at 3200 cm^{-1} mainly derived from the hydroxyl groups, and a significant increase in the area of the peak can also be found after HNO_3 treatment.

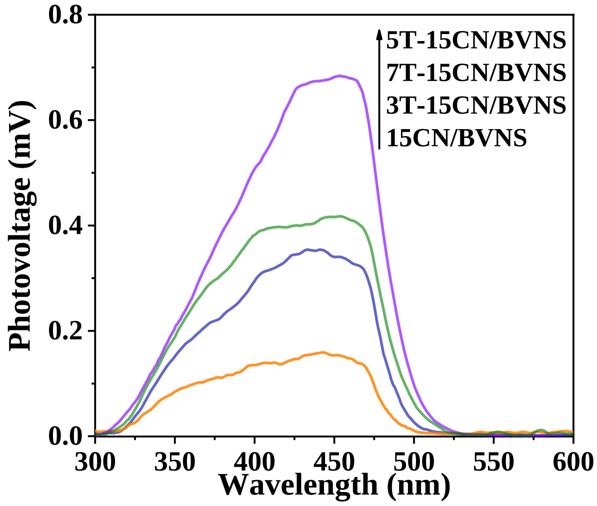

Figure S16 SS-SPS responses of 15CN/BVNS and yT-15CN/BVNS in N_2 atmosphere.

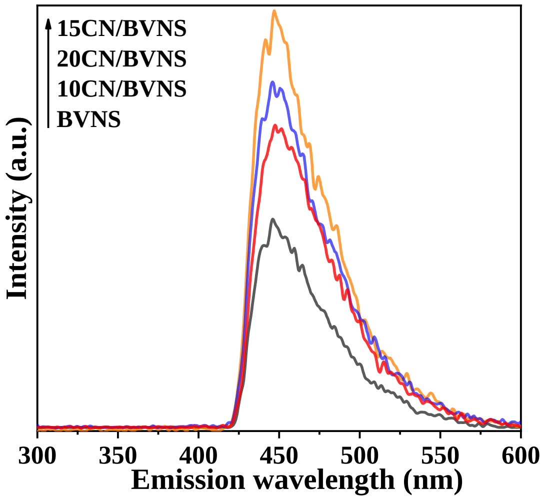

Figure S17 Fluorescence spectra related to the formed hydroxyl radicals of BVNS and xCN/BVNS.

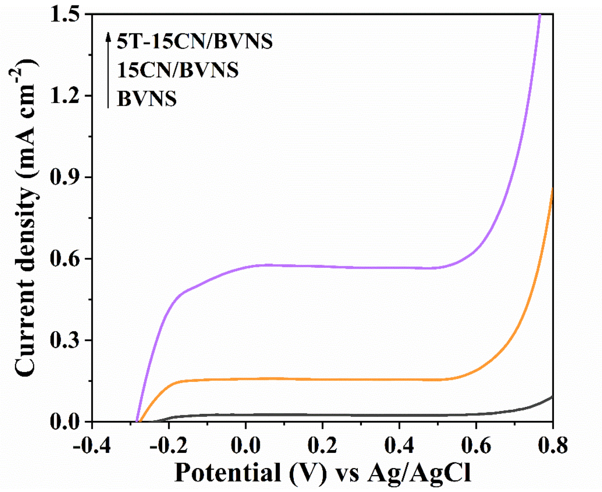

Figure S18 LSV curves of BVNS, 15CN/BVNS and 5T-15CN/BVNS in 0.2 M Na_2SO_4 electrolyte.

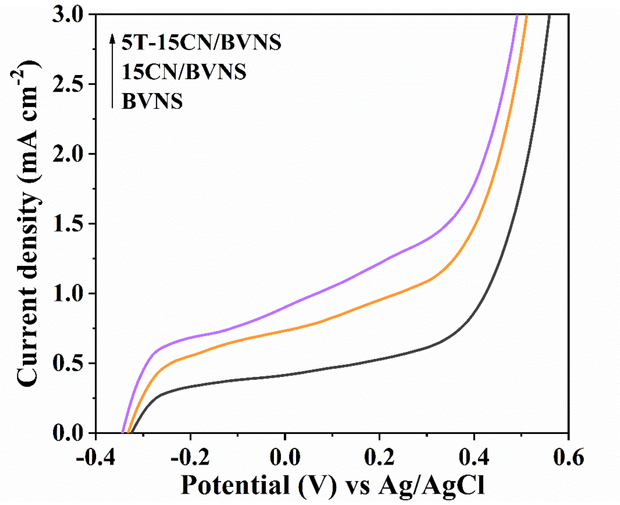

Figure S19 LSV curves of BVNS, 15CN/BVNS and 5T-15CN/BVNS in 0.2 M Na_2SO_4 electrolyte with 0.1 M Na_2SO_3 as a hole sacrificial agent.

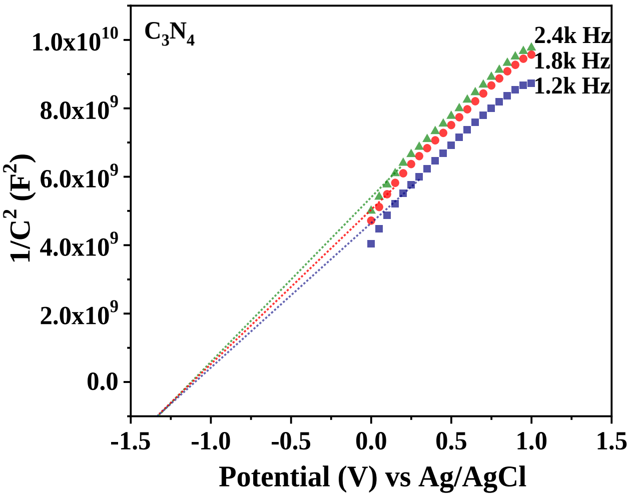

Figure S20 Mott-Schottky plots of the resulting CN collected at various frequencies versus the saturated Ag/AgCl reference electrode.

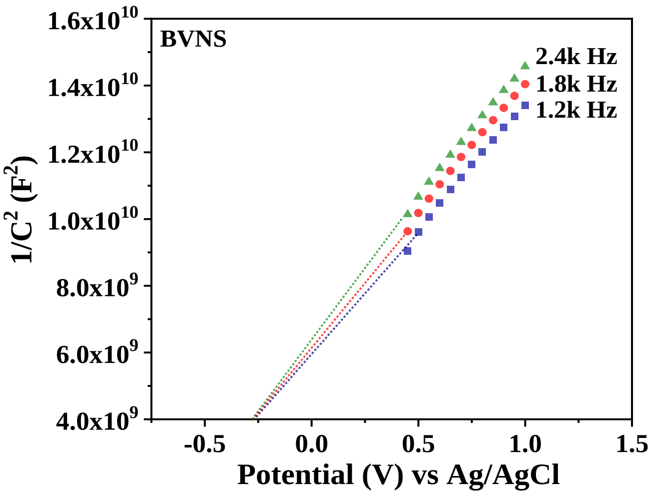

Figure S21 Mott-Schottky plots of the resulting BVNS collected at various frequencies versus the saturated Ag/AgCl reference electrode.

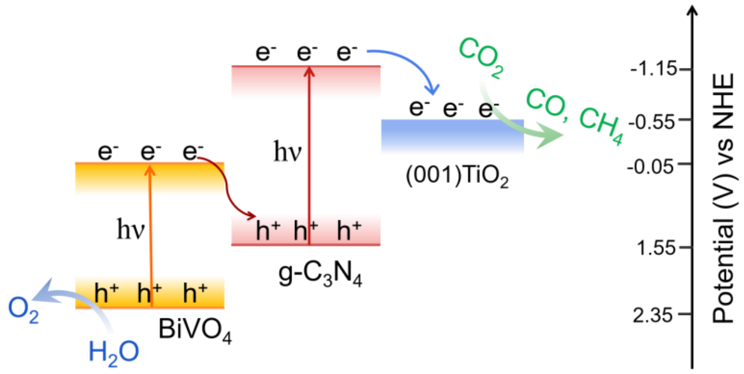

Figure S22 Illustration of energy band structures of the cascade Z-scheme heterojunction composed of BVNS, CN and T.

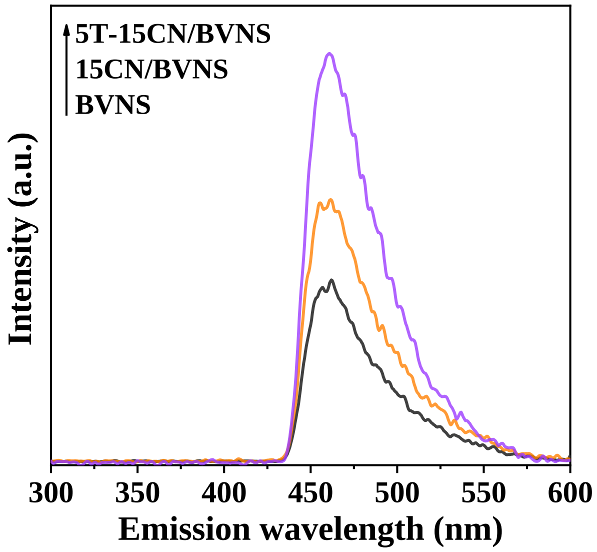

Figure S23 Fluorescence spectra related to the formed hydroxyl radicals of BVNS, 15CN/BVNS and 5T-15CN/BVNS under 405 nm irradiation.

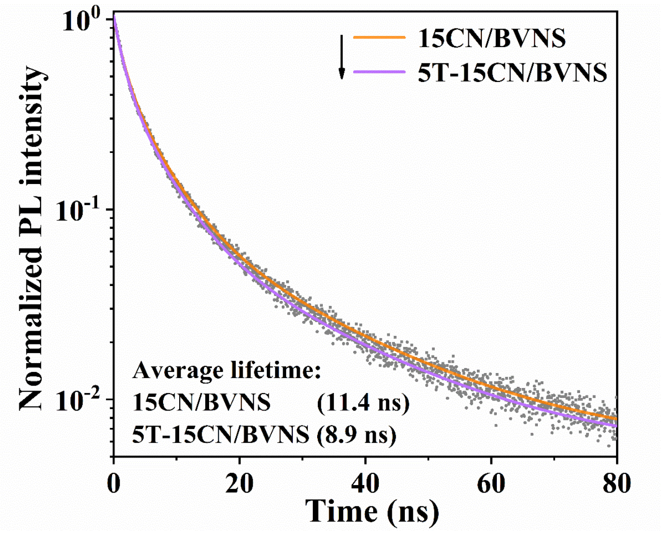

Figure S24 Time-resolved PL spectra with excitation wavelength of 355 nm of 15CN/BVNS and 5T-15CN/BVNS.

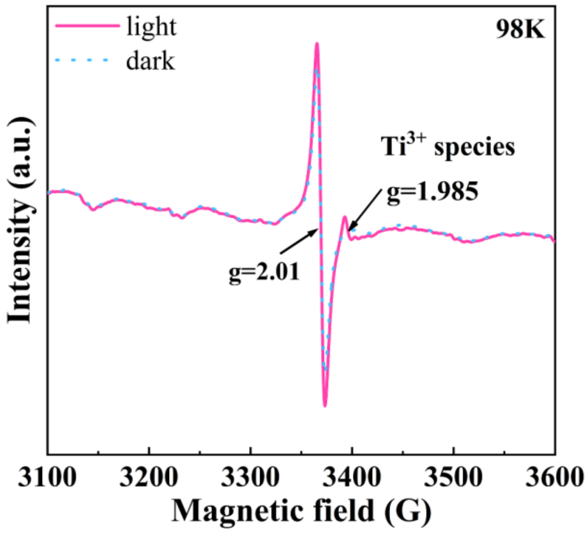

Figure S25 EPR spectra of 30T/CN heterojunction in dark and under visible-light irradiation at 98K (30 is the mass ratio percentage of T to CN).

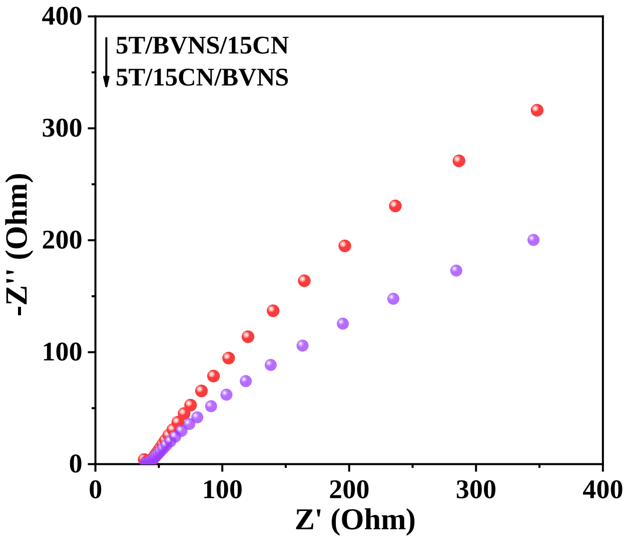

Figure S26 EIS spectra of 5T/15CN/BVNS and 5T/BVNS/15CN.

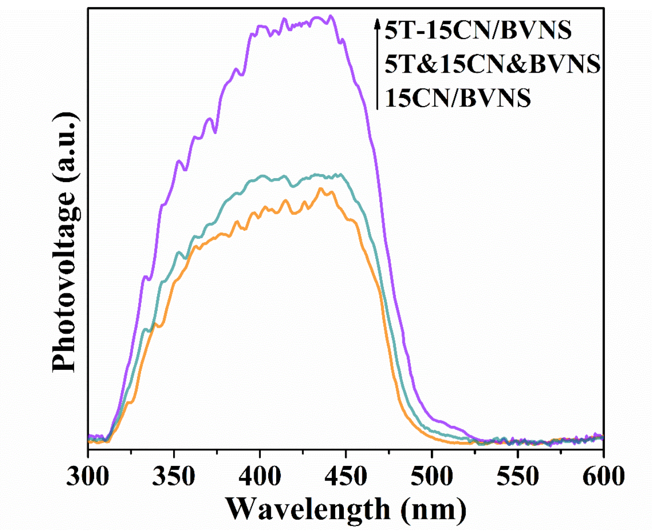

Figure S27 SS-SPS responses of 15CN/BVNS, 5T-15CN/BVNS and 5T&15CN&BVNS in N_2 atmosphere.

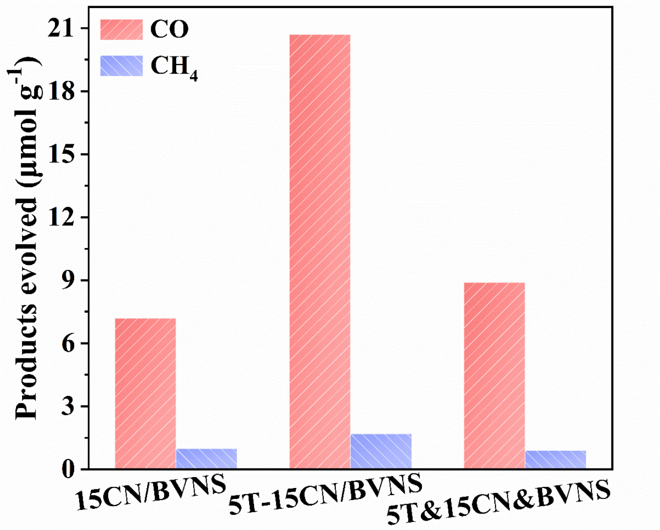

Figure S28 Photocatalytic activities for CO_2 reduction of 15CN/BVNS, 5T-15CN/BVNS and 5T&15CN&BVNS under visible-light irradiation for 4 h.

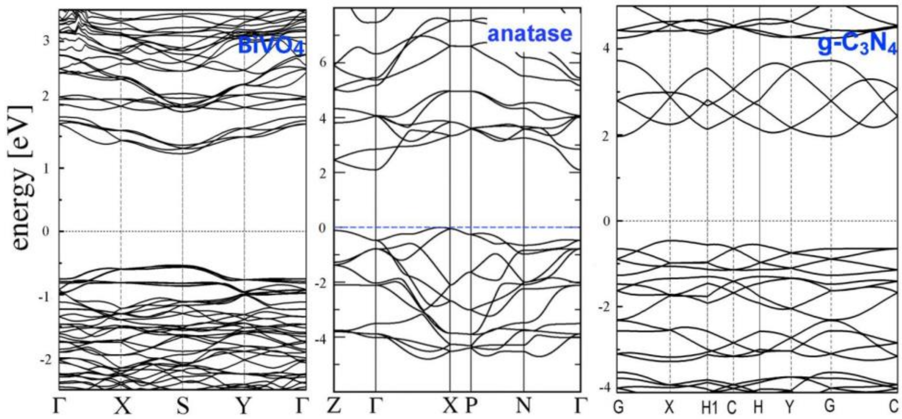

Figure S29 The band structures of $BiVO_4$, TiO_2 and g-C_3N_4, respectively.

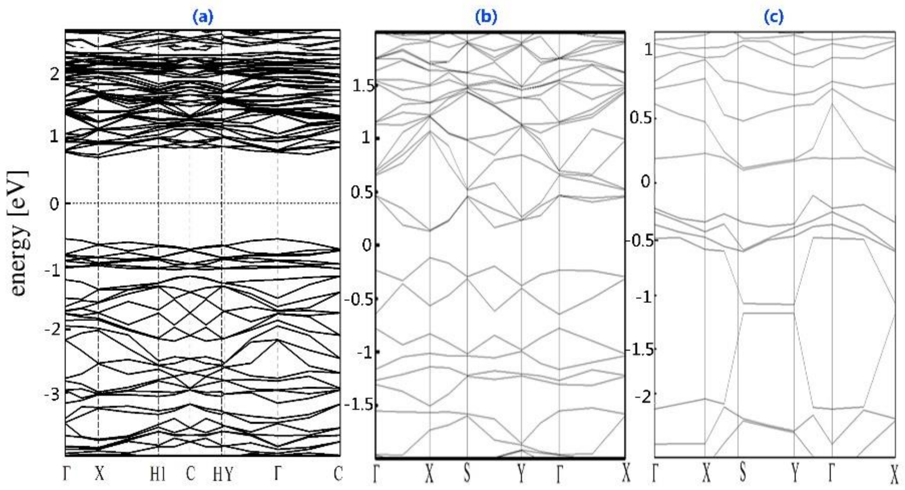

Figure S30 The band structures of CN/BVNS (a), T-CN/BVNS (b), T-CN_{2L}/BVNS (c), corresponding to the heterostructures (a)(b)(c) in Fig 6, respectively.

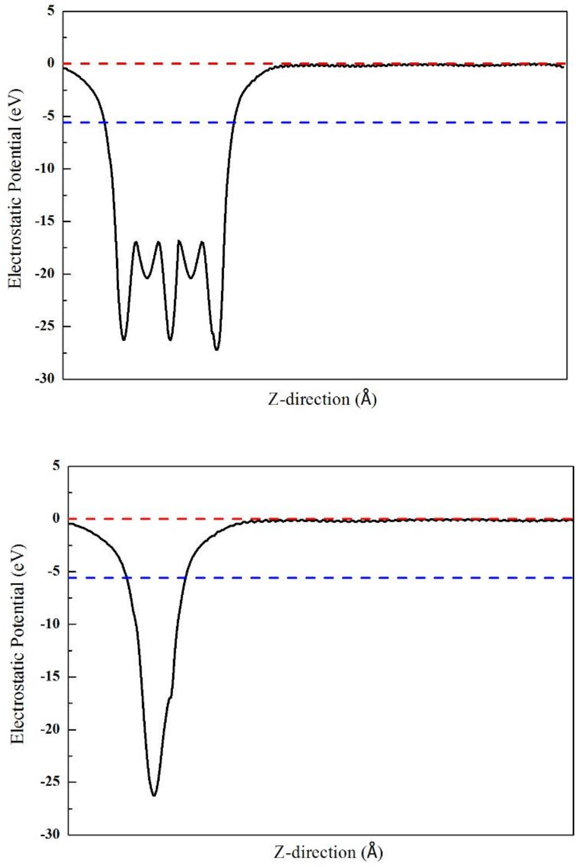

Figure S31 Work functions (i.e. electrostatic potentials) of monolayer g-C_3N_4 (top), and multilayer g-C_3N_4 (lower part). The work function stays at 5.85 eV.

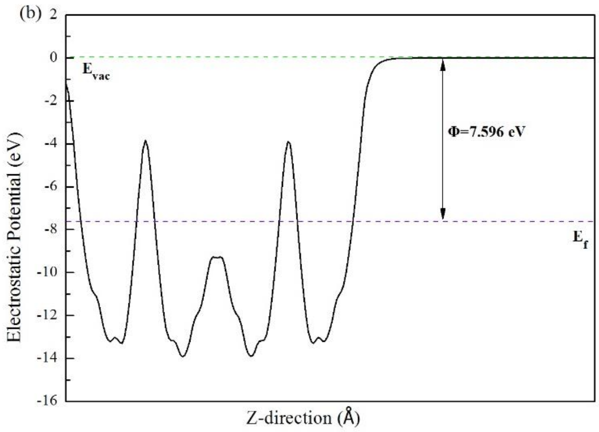

Figure S32 Work functions (i.e. electrostatic potentials) of $BiVO_4$ slab.

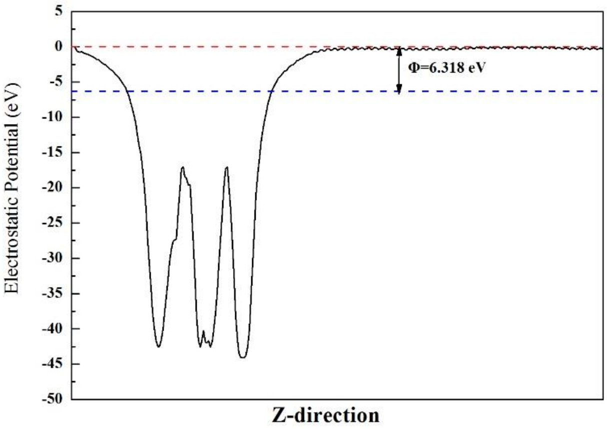

Figure S33 Work functions (i.e. electrostatic potentials) of anatase TiO_2 (001) slab.

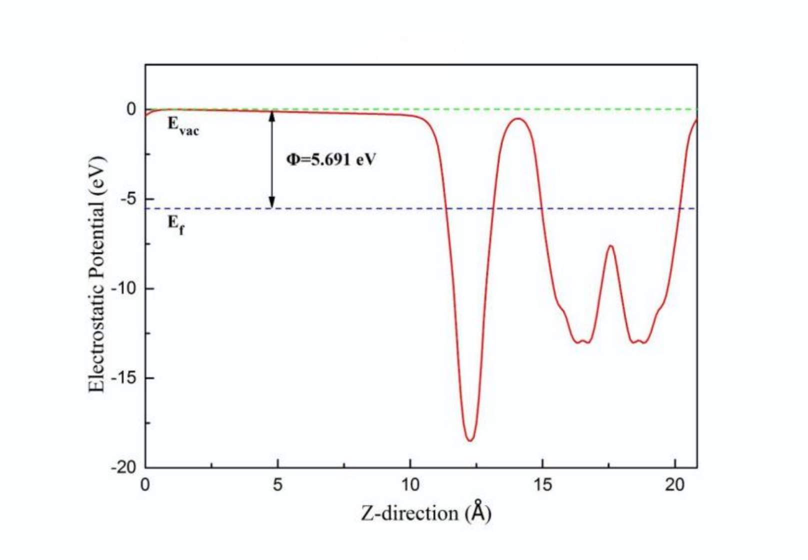

Figure S34 Work functions (i.e. electrostatic potentials) of CN/BVNS.

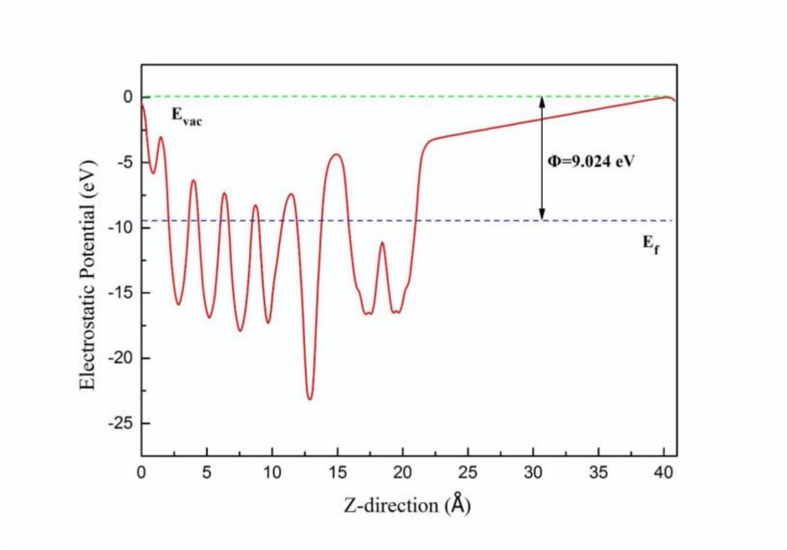

Figure S35 Work functions (i.e. electrostatic potentials) of T-CN/BVNS.

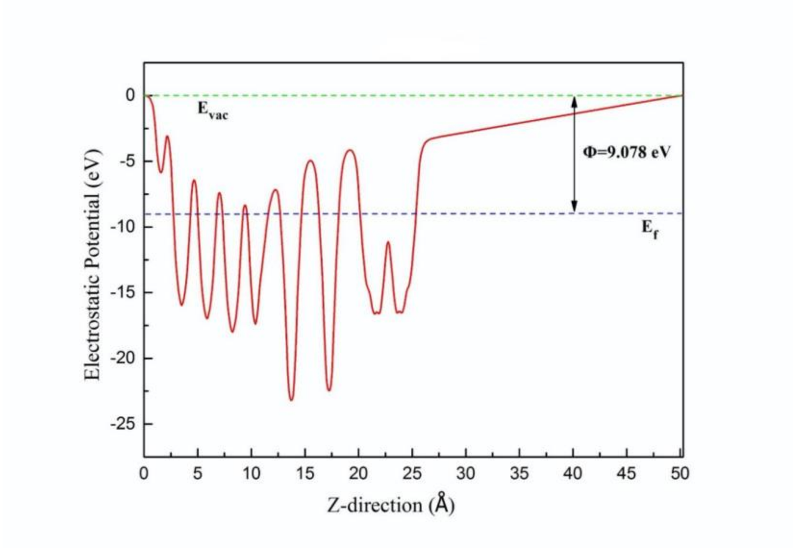

Figure S36 Work functions (i.e. electrostatic potentials) of T-CN_{2L}/BVNS.

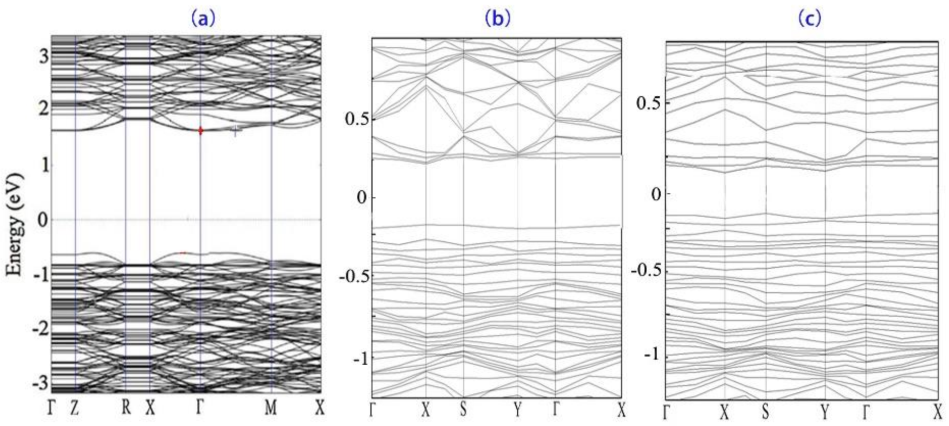

Figure S37 The band structures of Im-CN/BVNS (a), T-Im-CN/BVNS (b), T-Im-CN-Im/BVNS (c), corresponding to the heterostructures (e)(f)(g) in Fig 6, respectively.

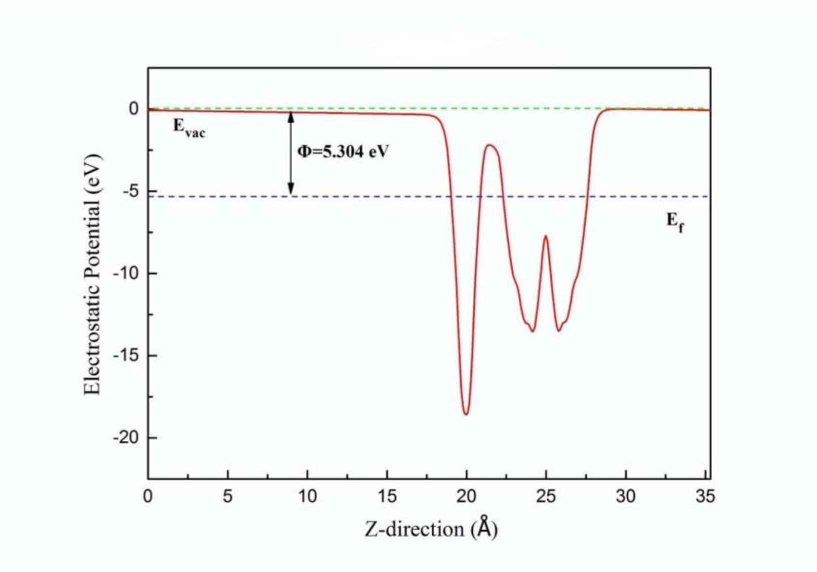

Figure S38 Work functions (i.e. electrostatic potentials) of Im-CN/BVNS.

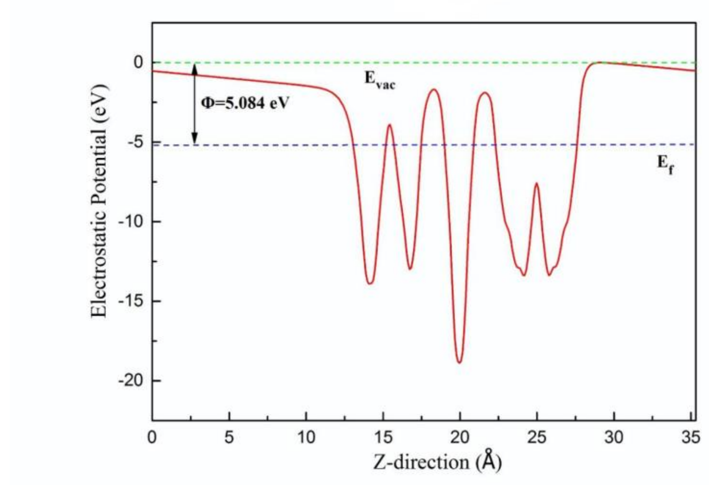

Figure S39 Work functions (i.e. electrostatic potentials) of T-Im-CN/BVNS.

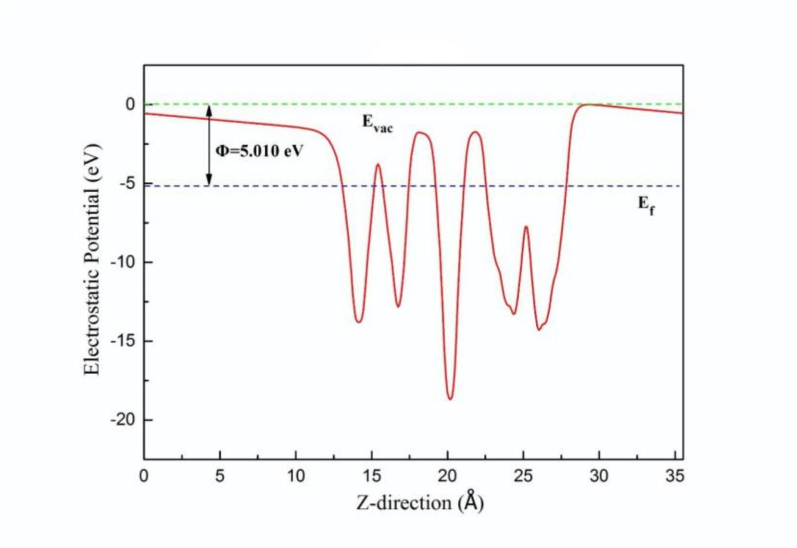

Figure S40 Work functions (i.e. electrostatic potentials) of T-Im-CN-Im/BVNS.

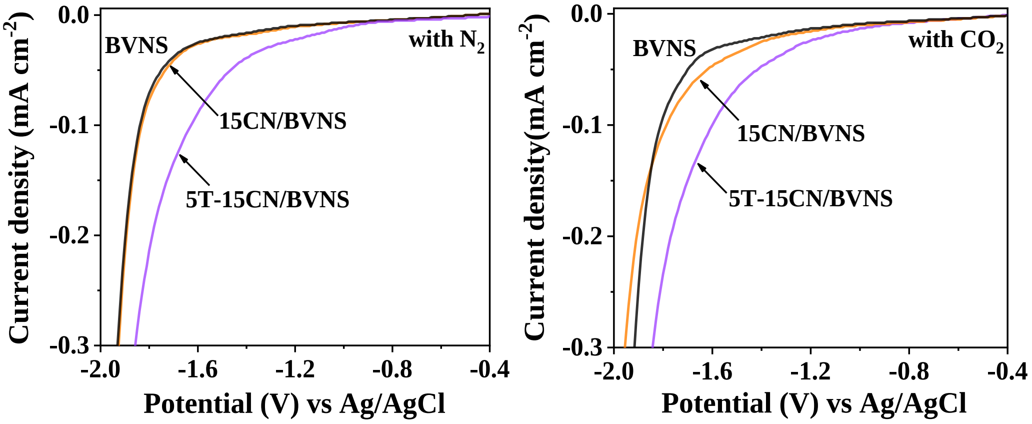

Figure S41 Electrochemical reduction curves in N_2-bubbled and CO_2-bubbled systems of BVNS, 15CN/BVNS and 5T-15CN/BVNS.

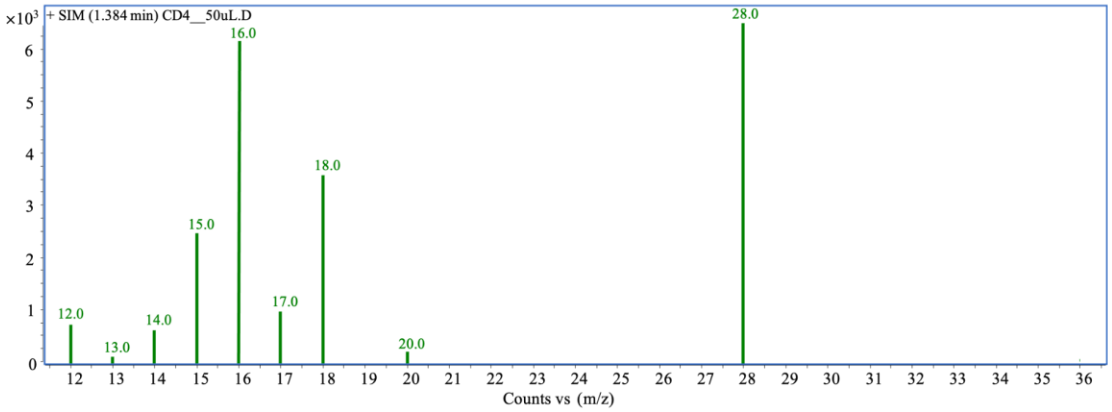

Figure S42 The GC-Mass analysis of photocatalytic CO_2 reduction products on 5T-15CN/BVNS after irradiation for 4 h with methanol as a hole scavenger with D_2O as a reductant. The major product CD_4 (m/z=20) and the related fragment CD (m/z =14), CD_2 (m/z =16), CD_3 (m/z =18) were detected apart from CD_2H (m/z =17), CDH (m/z =15) as well as CO (m/z=28).

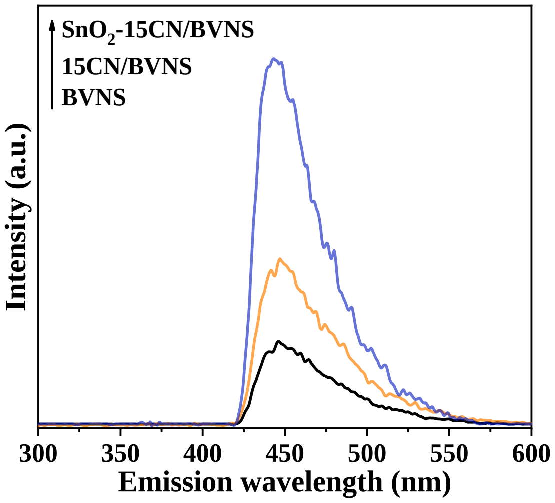

Figure S43 Fluorescence spectra related to the formed hydroxyl radicals under visible-light irradiation.

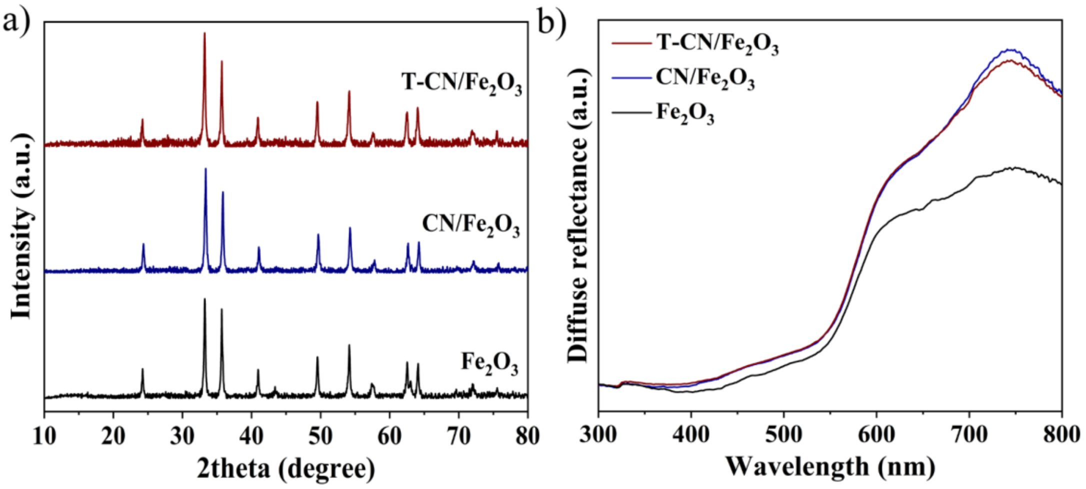

Figure S44 XRD patterns (a) and DRS spectra (b) of Fe_2O_3, CN/Fe_2O_3 and T-CN/Fe_2O_3.

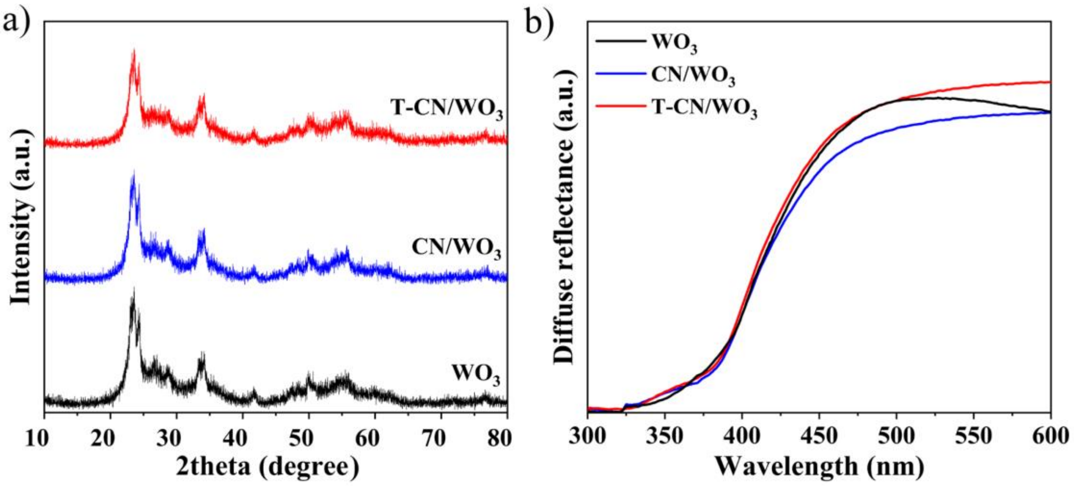

Figure S45 XRD patterns (a) and DRS spectra (b) of WO_3, CN/WO_3 and T-CN/WO_3.

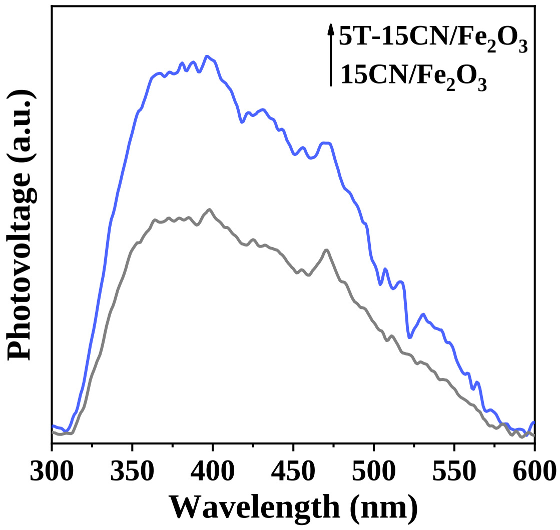

Figure S46 SPS responses of 15CN/Fe_2O_3 and 5T-15CN/Fe_2O_3.

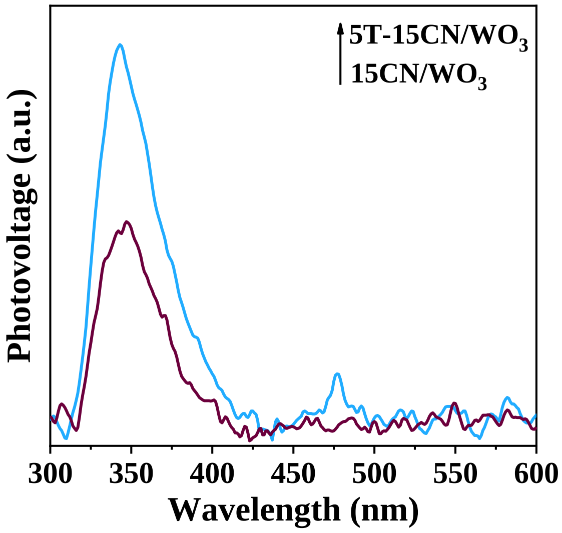

Figure S47 SPS responses of 15CN/WO_3 and 5T-15CN/WO_3.

Table S1 Comparison of photocatalytic CO_2 reduction performance of the reported $BiVO_4$-based photocatalysts in Z-scheme charge transfer.

Sample	CO evolution (μmol g^{-1} h^{-1})	Light source （W）	Ref.
5T-15CN/BVNS	5.18	300 W Xe lamp (λ ≥ 420 nm)	This work
15CN/BVNS	1.8	300 W Xe lamp (λ ≥ 420 nm)	This work
BVNS	0.29	300 W Xe lamp (λ ≥ 420 nm)	This work
ZnPc/BVNS	0.95	300 W Xe lamp (λ ≥ 420 nm)	[11]
$BiVO_4/C/Cu_2O$	3	300 W Xe lamp (λ ≥ 420 nm)	[12]
$BiVO_4$/Ru-complex/$(CuGa)_{1-x}Zn_{2x}S_2$	2.2	500 W Xe lamp (λ ≥ 420 nm)	[13]
$CoO_x/BiVO_4/RGO/Cu_2ZnGeS_4$	3.57	300 W Xe lamp (λ ≥ 420 nm)	[14]
$BiVO_4${010}-Au-Cu_2O	2.08	300 W Xe lamp (λ ≥ 420 nm)	[15]

Table S2 The fitted results of Nyquist plots for BVNS, 15CN/BVNS and 5T-15CN/BVNS.

Sample	Rs(Ω)	Rct(Ω)	CPE(F cm^{-2})
BVNS	45.39	2033	2.44E-5
15CN/BVNS	43.41	870.5	3.02E-5
5T-15CN/BVNS	41.91	436.1	3.64E-5

Table S3 The calculated surface charge transfer efficiency and photocurrent densities of BVNS, 15CN/BVNS and 5T-15CN/BVNS in 0.2 M Na_2SO_4 electrolyte (pH 6.8) at the applied potential of 0.2 V *versus* Ag/AgCl without and with 0.1 M Na_2SO_3, respectively.

Sample	J_{H_2O}(mA cm^{-2})	$J_{Na_2SO_3}$(mA cm^{-2})	Surface charge transfer efficiency (%)
BVNS	0.03	0.53	5.60
15CN/BVNS	0.15	0.94	15.90
5T-15CN/BVNS	0.57	1.20	47.50

References

[1] Y. T. Xiao, G. H. Tian, W. Li, Y. Xie, B. J. Jiang, C. G. Tian, D. Y. Zhao, H. G. Fu, *J. Am. Chem. Soc.* **2019**, *141*, 2508-2515.

[2] G. Kresse, J. Furthmiiller, *Comput. Mater. Sci*. **1996**, *6*, 15-50.

[3] G. Kresse, J. Furthmu, *Phys. Rev. B: Condens. Matter Mater. Phys*. **1996**, *54*, 11169-11186.

[4] P. E. Blochl, *Phys. Rev. B* **1994**, *50*, 17953-17979.

[5] J. P. Perdew, K. Burke, M. Ernzerhof, *Phys. Rev. Lett.* **1996**, *77*, 3865-3868.

[6] S. Grimme, J. Semiempirical, *Comput. Chem.* **2006**, *27*, 1787-1799.

[7] J. Cerdá, F. Soria, *Phys. Rev. B* **2000**, *61*, 7965-7971.

[8] J. Y. Xi, R. Jia, W. Li, J. Wang, F. Q. Bai, R. I. Eglitis, H. X. Zhang, *J. Mater. Chem. A* **2019**, *7*, 2730-2740.

[9] A. W. Sleight, H. Y. Chen, A. Ferretti, *Mater. Res. Bull*. **1979**, *208*, 1571-1581.

[10] J. K. Burdett, T. Hughbanks, G. J. Miller, J. W. Richardson, J. V. Smith, *J. Am. Chem. Soc.* **1987**, *109*, 3639-3646.

[11] J. Bian, J. N. Feng, Z. Q. Zhang, Z. J. Li, Y. H. Zhang, Y. D. Liu, S. Ali, Y. Qu, L. L.Bai, J. J. Xie, D. Y. Tang, X. Li, F. Q. Bai, J. W. Tang, L. Q. Jing, *Angew. Chem. Int. Ed.* **2019**, *58*, 10873-10878.

[12] C. Kim, K. M. Cho, A. Al-Saggaf, I. Gereige, H. T. Jung, *ACS Catal*. **2018**, *8*, 4170-4177.

[13] T. M. Suzuki, S. Yoshino, T. Takayama, A. Iwase, A. Kudo, T. Morikawa, *Chem. Commun*. **2018**, *54*, 10199-10202.

[14] A. Iwase, S. Yoshino, T. Takayama, Y. H. Ng, R. Amal, A. Kudo, *J. Am. Chem. Soc.* **2016**, *138*, 10260-10264.

[15] C. G. Zhou, S. M. Wang, Z. Y. Zhao, Z. S. Shi, S. C. Yan, Z. G. Zou, *Adv. Funct. Mater.* **2018**, *28*, 1801214.
